# A high-capacity cathode for rechargeable K-metal battery based on reversible superoxide-peroxide conversion

**DOI:** 10.1093/nsr/nwaa287

**Published:** 2020-11-27

**Authors:** Yu Qiao, Han Deng, Zhi Chang, Xin Cao, Huijun Yang, Haoshen Zhou

**Affiliations:** Energy Technology Research Institute, National Institute of Advanced Industrial Science and Technology (AIST), 1-1-1, Umezono, Tsukuba 305-8568, Japan; Center of Energy Storage Materials & Technology, College of Engineering and Applied Sciences, Jiangsu Key Laboratory of Artificial Functional Materials, National Laboratory of Solid State Microstructures, and Collaborative Innovation Center of Advanced Microstructures, Nanjing University, Nanjing 210093, China; Energy Technology Research Institute, National Institute of Advanced Industrial Science and Technology (AIST), 1-1-1, Umezono, Tsukuba 305-8568, Japan; Energy Technology Research Institute, National Institute of Advanced Industrial Science and Technology (AIST), 1-1-1, Umezono, Tsukuba 305-8568, Japan; Energy Technology Research Institute, National Institute of Advanced Industrial Science and Technology (AIST), 1-1-1, Umezono, Tsukuba 305-8568, Japan; Energy Technology Research Institute, National Institute of Advanced Industrial Science and Technology (AIST), 1-1-1, Umezono, Tsukuba 305-8568, Japan; Center of Energy Storage Materials & Technology, College of Engineering and Applied Sciences, Jiangsu Key Laboratory of Artificial Functional Materials, National Laboratory of Solid State Microstructures, and Collaborative Innovation Center of Advanced Microstructures, Nanjing University, Nanjing 210093, China; Energy Technology Research Institute, National Institute of Advanced Industrial Science and Technology (AIST), 1-1-1, Umezono, Tsukuba 305-8568, Japan

**Keywords:** electrochemistry, rechargeable battery, beyond Li-ion battery, long-term cycle life, high capacity cathode

## Abstract

As a promising low-cost energy storage device, the development of a rechargeable potassium-ion battery (KIB) is severely hindered by the limited capacity of cathode candidates. Regarded as an attractive capacity-boosting strategy, triggering the O-related anionic redox activity has not been achieved within a sealed KIB system. Herein, in contrast to the typical gaseous open K-O_2_ battery (O_2_/KO_2_ redox), we originally realize the reversible superoxide/peroxide (KO_2_/K_2_O_2_) interconversion on a KO_2_-based cathode. Controlled within a sealed cell environment, the irreversible O_2_ evolution and electrolyte decomposition (induced by superoxide anion (O_2_^−^) formation) are effectively restrained. Rationally controlling the reversible depth-of-charge at 300 mAh/g (based on the mass of KO_2_), no obvious cell degradation can be observed during 900 cycles. Moreover, benefitting from electrolyte modification, the KO_2_-based cathode is coupled with a limited amount of K-metal anode (merely 2.5 times excess), harvesting a K-metal full-cell with high energy efficiency (∼90%) and long-term cycling stability (over 300 cycles).

## Teaser

The reversible superoxide/peroxide (KO_2_/K_2_O_2_) interconversion has been originally realized in a rechargeable battery technology, which can be extended as an attractive capacity-boosting strategy for energy storage devices.

## INTRODUCTION

Enlarging the energy density is a universal and eternal topic for energy storage devices, especially for low-cost potassium-ion battery (KIB) technology, in which the limited specific capacities of the cathode seriously hinder its further development [1–6]. Due to the confined choices of cathode candidates, sluggish progress has been made on the cathode side of KIBs [7–9].

Introducing the O-related anionic redox activity into the cathode reaction has been regarded as a promising way to boost the specific capacity, e.g. Li/Na-O_2_ batteries and Li/Na-rich layered oxide cathodes [10–13]. Actually, as for the potassium-based system, the non-aqueous K-O_2_ battery relying on O-related pure anionic redox (the conversion between gaseous O_2_ and KO_2_) was originally introduced by Wu's team in 2013 [14], and has made remarkable progress with regard to the aspects of electrolyte stability and KO_2_/electrolyte interface in the past seven years [14–20]. However, the K-O_2_ battery would inevitably suffer from the intrinsic challenges of the typical gaseous open battery system [21,22]. For instance, during the practical evolution from K-O_2_ to K-air battery, the moisture and CO_2_ from the air would essentially influence both the cathode reaction pathway and anode stability [23], while carrying the cumbersome supplementary facility (either air purifier devices or an O_2_ storage cylinder) would add huge burden on improving energy density. In this case, there is still a long way to go to evolve the gaseous open battery system into a practical energy storage device, while controlling the redox among various solid phases within a typical sealed cell environment seems to be more practical [21,22,24,25], e.g. interconversion among superoxide, peroxide and oxide for KIB technology. Unfortunately, so far no attempt has been reported to successfully confine the high-energy-density O-related anionic redox reaction within a practical sealed cell environment in a KIB. The pioneering realization of the K-O_2_ battery has demonstrated that KO_2_ can be regarded as a thermodynamically stable compound for K-ion storage [14,26]. Furthermore, enlightened by the recently-arisen oxide/peroxide-based cathode reaction in lithium-related battery systems (Li_2_O_2_/Li_2_O interconversion) [24,25], we propose a cathode reaction that operates via superoxide/peroxide (KO_2_/K_2_O_2_) interconversion, in which a theoretical specific capacity of 377 mAh/g (based on the mass of preloaded KO_2_) can be expected at a thermodynamic equilibrium potential of 1.92 V vs. K/K^+^ [27]. In this proposed system, not only the traditional electrolyte stability issue (against superoxide anion, O_2_^−^) should be considered, but also the charge overpotential should be rationally controlled to restrain O_2_ evolution (corresponding thermodynamic equilibrium potential of OER is summarized in Supporting Data).

In this work, the proposed non-O_2_ reversible KO_2_/K_2_O_2_ interconversion has been successfully achieved by embedding KO_2_ into a catalytic ruthenium dioxide (RuO_2_) nanoparticles-loaded reduced-graphene-oxide (rGO) matrix. Demonstrated as the culprit for irreversible O_2_ evolution and electrolyte decomposition, the formation of a superoxide anion (O_2_^−^) has been restrained by the fabrication of a unique K-deficient K_1-x_O_2_-Ru intermediate state on the electrode/electrolyte interface during charging. As a result, the half-cell presents merely 0.2 V average round-trip overpotential and provides a reversible capacity of 300 mAh/g (based on the mass of preloaded KO_2_) during 900 cycles. Moreover, benefitting from electrolyte modification, the corresponding full-cells deliver superior cycling stability after being assembled with a limited amount of excess K-metal anode, which is remarkably competitive with other state-of-the-art KIB systems.

## RESULTS AND DISCUSSION

**Figure 1.**
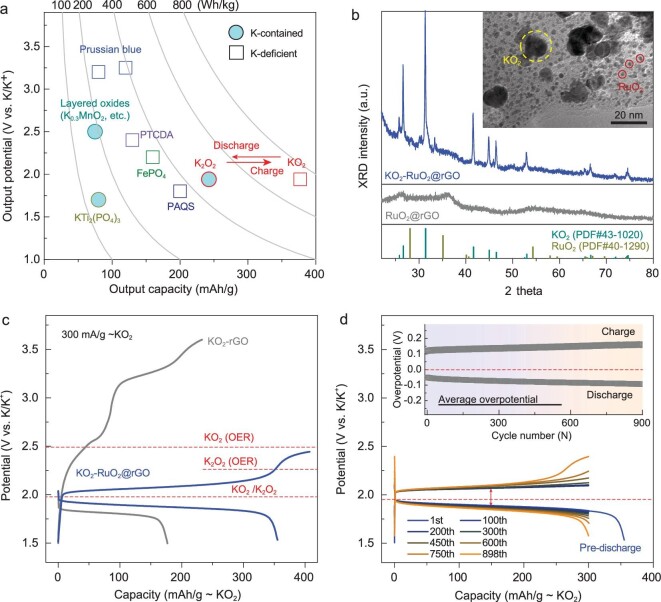
Characterizations and half-cell performance of the KO_2_-based cathode for KIBs. (a) Theoretical output potential, specific capacity and energy density for the KO_2_ and other typical K-ion battery cathodes. (b) XRD pattern and TEM images of KO_2_-RuO_2_@rGO cathode composites. The XRD pattern of KO_2_-rGO is shown for comparison. (c) Galvanostatic discharge/charge curves (the initial cycle) of KO_2_-rGO (gray traces) and KO_2_-RuO_2_@rGO (blue traces) cathodes, respectively. (d) Typical discharge/charge curves of the KO_2_-RuO_2_@rGO cathode collected in the half-cell (coupled with large excess amount of K-metal anode). The average overpotentials (vs. thermodynamics 1.94 V) upon charging and discharging are shown in the inset for clarity. The current density is fixed at 300 mA/g∼KO_2_.

In order to make fair comparisons with regard to cathode energy density in KIBs, the cathode candidates should be grouped into two categories (based on different pristine states): K-contained and K-deficient states (Fig. 1a and Table S1). As for the proposed KO_2_/K_2_O_2_ redox process, the pristine KO_2_ state is the K-deficient state, which has a prominent advantage over other candidates (Prussian blue cathode, etc.) [8,9,28–31]. Meanwhile, compared to other K-contained cathodes (layered oxides, etc.) [2,32,33], the corresponding K_2_O_2_ (K-contained discharged state) also presents large superiority on the aspect of energy density. As the cathode matrix, RuO_2_@rGO nano-composite is prepared via a typical microwave-hydrothermal method [34], in which the RuO_2_ nanoparticles (NPs, 2–3 nm in size, RuO_2_: 75 wt%) are homogeneously dispersed onto the rGO sheet (Fig. S1). After sufficient ball-milling procedure, KO_2_ is mixed with the RuO_2_@rGO matrix (KO_2_: 60 wt%), and KO_2_-RuO_2_@rGO cathode composite can be harvested, which is identified by X-ray diffraction (XRD) characterization (Fig. 1b). Visual transmission electron microscopy (TEM) images clearly show that KO_2_ NPs (10–15 nm in size) are well dispersed and embedded within the RuO_2_@rGO matrix (inset Fig. 1b and Fig. S2). In the bulk cathode, the stacking of 2D rGO sheets would finally lead to an interlaced 3D network, in which the conductive pathway can be well guaranteed by the enfolding mode of KO_2_ and rGO substrate, while the tiny catalytic RuO_2_ NPs intimately surround the KO_2_ active materials, and sufficiently enrich their contact sites. The role of RuO_2_ will be further interpreted subsequently.

Within a half-cell, the as-prepared KO_2_-based cathode is coupled with a huge excess amount of K-metal (merely for cathode assessment); the electrolyte issue will be discussed in the following full-cell section. As for RuO_2_-free KO_2_-rGO cathode (gray trace, Fig. 1c), the discharge process is initially conduced for KO_2_ reduction, while the subsequent oxidization/charging potential rapid climbs and then surpasses the thermodynamic oxygen evolution reaction (OER) potentials of both K_2_O_2_ and KO_2_ (2.2 V and 2.48 V, respectively). A very different electrochemical behavior can be observed on the KO_2_-RuO_2_@rGO cathode (blue trace, Fig. 1c), in which a long flat plateau can be obtained with an average potential of 1.88 V vs. K/K^+^ (0.06 V overpotental vs. thermodynamic potential) and a specific capacity of 355.6 mAh/g (94.3% depth-of-discharge vs. theoretical capacity). In order to control the reduction process, the cutoff voltage has been set at 1.5 V, at which the inactive RuO_2_-rGO substrate would not participate in the electrochemical redox process (Fig. S3). During subsequent charging, a plateau can be harvested with tiny polarization during the initial depth of 300 mAh/g. While, at the end of charging, the polarization phenomenon can be ascribed to the overcharge and OER. Turning to cycling performance (Fig. 1d), after a pre-discharging procedure, the KO_2_-RuO_2_@rGO cathode presents long-term cycling stability with a cutoff charge-depth at 300 mAh/g (to avoid undesired OER). After 750 cycles, obvious potential polarization can be observed at the end of charging, and the final charge potential touches the ‘OER redline’ (K_2_O_2_ OER potential: 2.2 V) at the end of the 750th cycle. During long-term cycling, the discharge/charge overpotential has been effectively restrained (Fig. 1d, inset), resulting in an average round-trip efficiency of 88.9% at a large current rate of 300 mA/g. Taking comprehensive assessment aspects into consideration (current density, cycling stability, specific capacity, energy/coulombic efficiency, etc.), the performance of the currently-introduced KO_2_-based cathode delivers considerable improvements beyond other reported cathode candidates in KIBs (Table S2). Moreover, the mass loading of KO_2_ is controlled around 3.0–4.0 mg/cm^2^, thus, the areal specific capacity of the current cathode can reach 0.9–1.2 mAh/cm^2^, which is indeed competitive even compared with typical K-O_2_ open battery systems.

**Figure 2.**
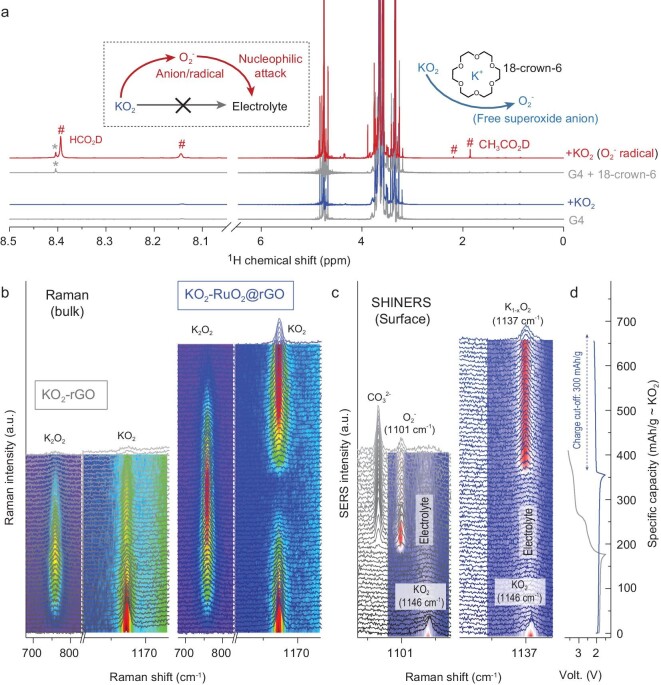
The redox behavior of KO_2_-based cathodes. (a) ^1^H NMR spectra of D_2_O-extracted G4-based electrolyte samples. After aging with KO_2_ powder (one week), the parasitic products are demonstrated by the comparison with corresponding KO_2_-free blank sample (gray traces). 18-crown-6 is employed to extract the K-ion from KO_2_, and induces the formation of a free O_2_^−^ anion. (b) Typical bulk-sensitive operando Raman spectra and (c) surface-sensitive surface-enhanced operando SHINERS spectra observed on KO_2_-rGO and KO_2_-RuO_2_@rGO cathodes recorded during initial galvanostatic cycle. The capacity-dependent spectra are offset, and the related spectral contour plots are shown for clarity. (d) Galvanostatic curves during operando observation.

Before systematically analyzing the electrochemical redox behavior, the difference between the superoxide (KO_2_) and superoxide anion (O_2_^−^) should be emphasized in advance. Typically, within non-aqueous metal-O_2_ batteries, the O_2_ molecule is initially reduced to an O_2_^−^ anion (oxygen reduction reaction, ORR), which would carry out a strong nucleophilic attack on organic electrolyte components, resulting in electrolyte decomposition [35,36]. After being combined with K-ion and deposit as a solid state (KO_2_ superoxide compound), the nucleophilic character can be essentially restrained [17,20,26]. Based on the nuclear magnetic resonance (NMR) observations on ether solvent (tetraethylene glycol dimethyl ether, TEGDME, G4), after aging with KO_2_ powder for one week, no obvious decomposition can be observed (blue trace, Fig. 2a). Once trace 18-crown-6 is added to extract the K-ion, the superoxide (KO_2_) would dissolve and convert into the relative free anion state (O_2_^−^) [17]. Without the stabilization of K-ion, the O_2_^−^ (radical) induced electrolyte decomposition, resulting in the observation of formate and acetate (8.39 and 1.92 ppm), respectively (red trace, Fig. 2a) [36]. In this case, as for the superoxide-involved battery systems, the solubility of superoxide in the electrolyte system should be strictly controlled (Fig. S4). In other words, as an ‘Achilles’ heel’, the superoxide anion (O_2_^−^) is an unescapable discharge intermediate product in typical metal-O_2_ batteries during ORR [37]. However, upon KO_2_/K_2_O_2_ interconversion process, the production of O_2_^−^ can be reasonably eradicated, if we protect KO_2_ against further oxidation during charging (limit the redox between KO_2_/K_2_O_2_, but do not extend to O_2_/KO_2_). In summary, as the chief culprit for both electrolyte decomposition and irreversible O_2_ evolution, the formation of O_2_^−^ should be critically restrained, which is the core mission for the achievement of reversible KO_2_/K_2_O_2_ interconversion.

Operando Raman spectroscopy is firstly employed to reveal the electrolyte redox process of KO_2_-based cathodes (Fig. 2b), in which the skin depth of typical Raman (0.1–5.0 μm related to electronic conductivity and magnetic permeability) can be regarded as a bulk measurement as the particle size of KO_2_ lies between 10–15 nm. As assigned in Fig. S5, the sharp peak located at 1146 cm^−1^ indicates the O-O stretch within the KO_2_-preloaded cathode at the open circuit voltage (OCV) state [38]. The spectra collected from the KO_2_-rGO and KO_2_-RuO_2_@rGO cathodes share a similar variation trend upon the initial discharging processes (around 175 mAh/g), during which the reduction of superoxide peak is accompanied by the growth of the peroxo-related one (at 759 cm^−1^) [39]. However, upon subsequent charging on KO_2_-rGO (left group, Fig. 2b), the peroxide has not been totally oxidized, nor the superoxide restored, which indicates an irreversible redox process. As a comparison, on the KO_2_-RuO_2_@rGO cathode (right group, Fig. 2b), symmetrically reversible variation trends can be clearly observed for both peroxide consumption and superoxide restoration, which indicates the achievement of reversible KO_2_/K_2_O_2_ interconversion. The corresponding capacity-dependence of Raman peak intensities and offset Raman spectrum are shown in Figs S6 and S7 for clarity. Strictly speaking, due to the relationship between skin depth and absorbance, operando Raman intensity variation can be regarded as a general quantification analysis, but not a rigorous one. In this case, by the combination of ‘double-check’ titrations and a gas chromatograph-mass spectrometer (GC-MS) characterization, we develop a more precise quantitative method [25], and the results reprove the reversible interconversion between KO_2_ and K_2_O_2_ during cycling on the KO_2_-RuO_2_@rGO cathode (Figs S8 and S9).

Continuously, we employ *insitu* shell-isolated nanoparticle-enhanced Raman spectroscopy (SHINERS) [40], a surface-sensitive operando observation (probe depth: ∼1.5 nm), to investigate the behaviors on the electrode surface/interface, which are essentially helpful to deeply reveal the electrochemical redox processes (Fig. 2c). Regardless of the broad peak around 1150 cm^−1^ assigned to the electrolyte (consistently existing during discharge/charge), a sharp peak at 1146 cm^−1^ (O–O stretch in KO_2_) can be observed at the pristine state of each KO_2_-preloaded cathode, which rapidly disappears at the initial stage of discharging. This indicates the surface of KO_2_ NPs has been reduced to K_2_O_2_, which shields the signal from the bulk KO_2_ core. While, from the beginning of charging, a sharp difference can be observed between KO_2_-rGO (left pattern, Fig. 2c) and KO_2_-RuO_2_@rGO (right pattern, Fig. 2c) cathodes. On the surface of the KO_2_-rGO cathode, the peak located at 1101 cm^−1^ gradually increases during charging, which is assigned to the adsorbed superoxide anion species (Fig. S5) [37]. Simultaneously, the appearance of the carbonate-related peak (at 1063 cm^−1^) can be rationally ascribed to the superoxo-induced electrolyte degradation [41]. While, on the KO_2_-RuO_2_@rGO cathode surface, a new peak located at 1137 cm^−1^ appears and rapidly becomes saturated at the initial stage of charging. In principle, at the very beginning of charging, the K_2_O_2_ (formed upon discharging) on the surface lose a K^+^ and are oxidized to the form of superoxo species. Without RuO_2_, surface K_2_O_2_ directly evolved into free superoxide anion, which presents strongly nucleophilic properties and can easily be further decomposed. In the RuO_2_-contained condition, the surface species can be rationally assigned to superoxide rather than superoxide anion, since it demonstrates a more combining state than a free anion state. Explained by Raman-related language, the peak located at 1101 cm^−1^ (free anion state, RuO_2_-free condition) indicates a larger O–O bond distance than the 1137 cm^−1^ peak observed in the RuO_2_-contained condition [38,39,42,43]. In this case, the interaction between K^+^ cation and superoxo-anion presents much more strongly at 1137 cm^−1^ (RuO_2_-contained condition), and close to the fully-compound state (KO_2_, 1146 cm^−1^) [37]. In other words, rather than oxidize to a free anion state upon charging, the KO_2_-RuO_2_@rGO cathode can be protected by a moderate K-deficient superoxide compound state (K_1-x_O_2_ state), a more stable and mild intermediate state than nucleophilic anion (O_2_^−^). Moreover, the electrolyte decomposition and carbonate formation cannot be observed on the KO_2_-RuO_2_@rGO cathode during charging, which is consistent with the moderate property of K-deficient K_1-x_O_2_ state.

**Figure 3.**
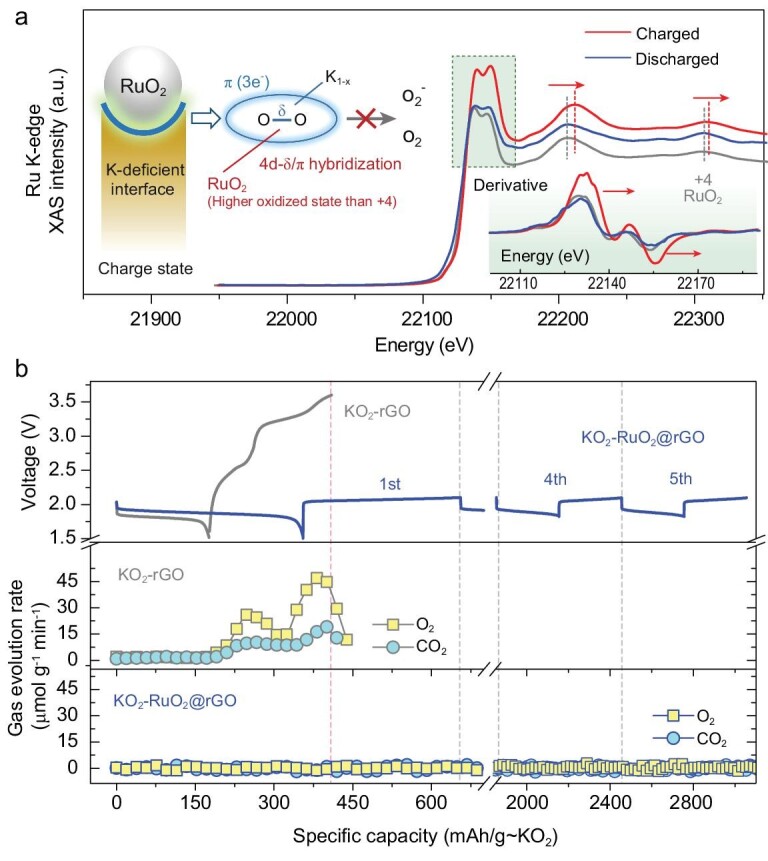
Analysis on KO_2_-based cathodes. (a) Ru K-edge hard XAS spectra collected from the KO_2_-RuO_2_@rGO cathode at discharged (blue trace) and charged (gray trace) states, respectively. The reference spectrum of RuO_2_ (+4) is shown for comparison. Corresponding first derivate plots in XANES region are shown in the inset for clarity. (b) Evolution rates of gaseous O_2_ and CO_2_ upon cycling collected on KO_2_-rGO (gray blocks, the first cycle) and KO_2_-RuO_2_@rGO (blue blocks, initial five cycles) cathodes.

Based on the surface investigations and related comparison, the RuO_2_ plays an important role for the as-obtained K-deficient superoxide state during charging. Hard X-ray absorption spectroscopy (XAS) is employed to further investigate the role of RuO_2_ (Fig. 3a). Compared with the K-edge Ru spectrum collected from RuO_2_ standard, the discharged state stays on Ru^4+^, since we cannot observe any obvious difference on near edge (XANES) nor extended region (EXAFS). However, at charged state, the Ru K-edge peaks shift towards higher energy, which can be attributed to the oxidation of Ru^4+^ into a higher valence state. The first derivative plot of XANES region (inset Fig. 3a) clearly shows the peaks shift. Due to the negligible charge capacity contribution from Ru^4+^ on KO_2_-free RuO_2_@rGO cathode (Fig. S3), the RuO_2_ would not directly participate in the charge compensation, but there still exists an interaction between RuO_2_ and surface KO_2_, resulting in the formation of a surface K-deficient K_1-x_O_2_ phase. On the aspect of molecular orbital, the covalency state between 4d transition metal (e.g. Ru) and peroxo-like (O_2_)^n−^ species has been reported to enhance the stability of the O-related anionic redox process via orbital hybridization (4d-σ^*^ remixing) and reductive coupling formation [44,45]. Moreover, a similar intermediate phase has been reported within the Li-deficient Li_2-x_O_2_ phase, which is assumed to prevent peroxide against OER decomposition [25,46]. In this case, combining the systematical information harvested from Raman (formation of the K-deficient superoxide phase) and XAS (interaction with Ru^4+^), we rationally conclude that the formation of a K-deficient K_1-x_O_2_-Ru surface phase prevents the further de-potassiation and O_2_^−^ formation, while, without the stabilization from RuO_2_, the unstable intermediate K-deficient superoxide phase would immediately turn to anion state during subsequent charging. The electrochemical redox process is schematically illustrated in Fig. S10. Moreover, without the formation of the K-deficient K_1-x_O_2_-Ru surface phase, a free O_2_^−^ anion would either suffer from further oxidation or attack the electrolyte, resulting in the release of both O_2_ and CO_2_, which was proven by *insitu* differential electrochemical mass spectroscopy (DEMS, Fig. 3b). As a comparison, O_2_ evolution cannot be observed on the KO_2_-RuO_2_@rGO cathode during five reversible cycles. This negative gas evolution coincides well with the reversible KO_2_/K_2_O_2_ redox process, which was previously demonstrated by both electrochemical and spectroscopic evidence.

**Figure 4.**
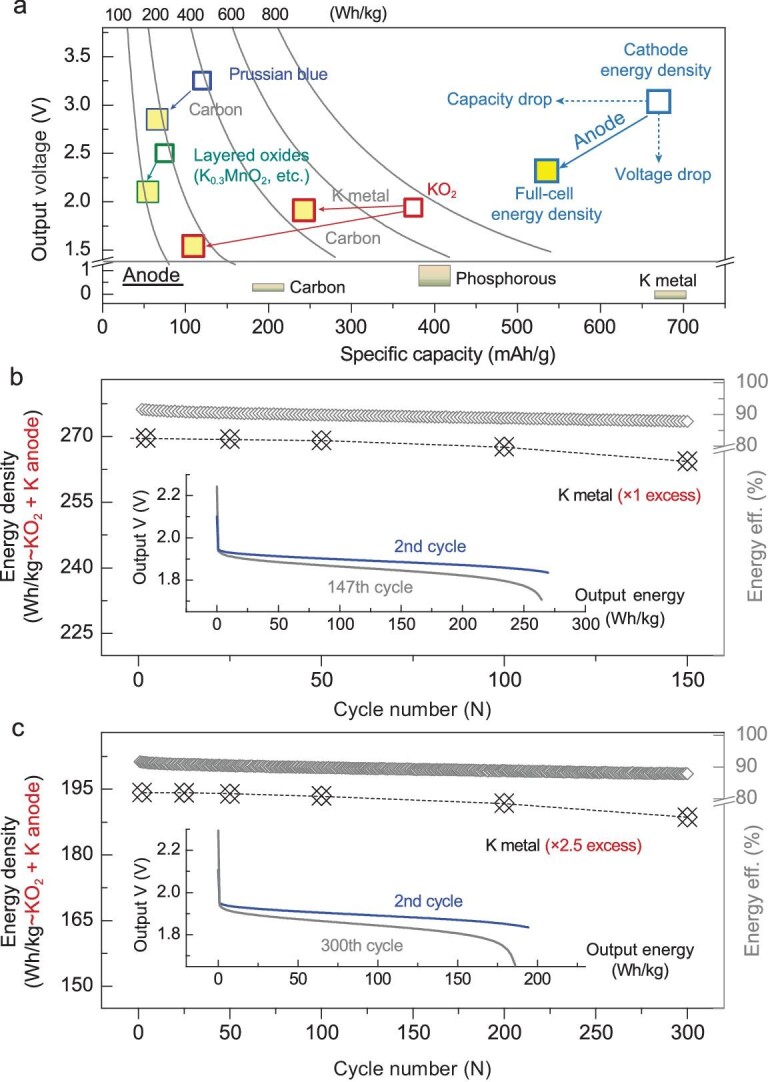
Cycling performance of related full-cells assembled with limited excess amount of K-metal anode. (a) Theoretical output potential/voltage, specific capacity and energy density for various cathode candidates (hollow blocks) and corresponding full-cell systems (yellow-filled blocks). The drop of cathode energy density in the full-cell is ascribed to the coupling of the anode. (b and c) Full-cells performance assembled with limited excess amount of K-metal anodes: (b) 100% excess and (c) 250% excess. Full-cell output energy densities (fairly calculated by the mass of both KO_2_ within the cathode and K-metal anode) are harvested by the integration of corresponding galvanostatic discharge curves. The round-trip energy efficiency indicates an assessment of cell polarization.

Finally, we want to further extend the currently-introduced cathode KO_2_/K_2_O_2_ redox reaction from proof-of-concept half-cell stage into the full-cell level. As shown in Fig. 4a, after being coupled with different anode candidates (carbon and K-metal, etc.) [47–50], the full-cell energy density would suffer from an obvious drop versus corresponding cathode energy density. The related conversion and calculation procedures on full-cell energy densities are illustrated in Fig. S11. In order to boost the full-cell energy density, we employ the most challenging K-metal anode for full-cell fabrication, and assemble the full-cell with limited amount of excess K-metal as anode (Fig. 4b and c). Relatively speaking, the K-contained cathodes (layered oxides, etc.) present as being more suitable to couple with K-deficient anodes (graphitic and phosphorus materials, etc.), in which the K-ion stored in the cathode would meet the ion transfer. While, as for K-deficient cathodes (Prussian blue, TiS_2_, organic cathodes, O_2_ cathode and KO_2_ herein, etc.), the K-contained metallic potassium would be the priority anode candidate. The electrolyte modification is remarkably important for enhancing the reversibility and efficiency of the K-metal anode. Benefitting from the positive role of classic bis(fluorosulfonyl) imide (FSI) anion [17], we further introduce a trace amount (5 wt%) of fluoro-ether additive to strengthen the stability of the K-metal surface (e.g. solid-state-interface) in the ether-based electrolyte system, resulting in a K–K symmetric cell with 1000 hours long-term plating/stripping stability (Fig. S12) and K-Cu half-cells performing high coulombic efficiency (CE% > 99%) before ∼250 cycles (Fig. S13). Herein, we do not carry out a deep investigation/characterization on the K-metal anode side, but provide the performance of fluorinated ether/carbonate candidates as a promising additive towards K-metal protection (Fig. S14). After introducing the highly-efficient K-metal system, we assemble the full-cell with merely 100% excess amount of K-metal anode (Fig. 4b). Due to the utilization of capacity-cutoff cycling mode (same cutoff depth as half cell: 300 mAh/g∼KO_2_), the cycle life and round-trip energy efficiency present as the most important parameters (Fig. S15). As a result, based on a fair calculation, the initial output energy density of the current full-cell reaches 270 Wh/kg (taking the mass of both KO_2_ and K-metal into consideration). Meanwhile, due to the effective restraining of potential polarization, the average energy efficiency remains at around 90% during 147 reversible cycles. After enlarging the excess amount of K-metal anode to 250%, reversible cycling can be achieved over 300 cycles with considerable round-trip efficiency (Fig. 4c).

## CONCLUSION

In conclusion, for the first time, we realized the reversible redox of superoxide/peroxide (KO_2_/K_2_O_2_) in potassium-ion battery cathode systems with a considerable specific capacity (300 mAh/g∼KO_2_), tiny potential polarization (∼0.2 V round-trip overpotential), high current rate (300 mA/g) and long-term cycling stability (around 900 cycles in the half-cell). By embedding the KO_2_ into the RuO_2_@rGO matrix, the reversible KO_2_/K_2_O_2_ interconversion was confined within O_2_^−^/O_2_-free region, which was proven by systematically *in/exsitu* quantitative/qualitative characterizations. Moreover, based on bulk/surface-sensitive spectroscopic observations, we originally demonstrated the formation of a TM-covalent K-deficient superoxide intermediate surface phase (K_1-x_O_2_-Ru), which protected the cathode against irreversible O_2_^−^/O_2_ evolution upon charging. In addition, benefitting from electrolyte modification, we coupled the KO_2_-based cathode with limited excess amount of K-metal anode, and the full-cell system also performed with high output energy density and superior cycling stability. Based on fair comparison with other cathode and full-cell candidates for KIBs, the improvements of the current system not only lie on high energy density, but also energy efficiency, high-rate performance and long-term cyclability. We believe the first successful achievement of reversible superoxide/peroxide interconversion cathode reaction in a KIB will stimulate the development of O-related anionic redox reactivity for the fabrication of high-energy-density rechargeable battery devices.

## MATERIALS AND METHODS

### Electrolytes and cathode preparations

Tetra ethylene glycol dimethylether (TEGDME, G4), 1,1,2,2-tetrafluoroethyl 2,2,3,3-tetrafluoropropyl ether and bis(2,2,2-trifluoroethyl) carbonate (Sigma Aldrich, >99%) were dried over freshly activated 3 Å and 4 Å molecular sieves for several days. Potassium bis(fluorosulfonyl)imide (KFSI, purity of >98%) and potassium bis(trifluoromethane) sulfonamide (KTFSI, purity of >98%) salts were purchased from Tokyo Chemical Industry Co., Ltd., and dried by heating under a vacuum in a 80°C oven overnight. Electrolyte was prepared and stored in a glove box under Ar atmosphere. The detailed electrolyte components: 0.5 M KTFSI, 1.0 M KFSI, 5 wt% fluorinated ether/carbonate additives. The water concentration in the electrolyte measured by Karl Fischer titration was ∼3 ppm.

For the RuO_2_@rGO based matrix, graphene oxide (GO) was purchased from Nanjing XFNANO Materials Tech Co., Ltd, and the preparation procedure of rGO-supported ultrafine RuO_2_ NPs was similar to the previously reported microwave-hydrothermal synthesis method [34]. The RuO_2_ loading in the RuO_2_@rGO nano-composites was estimated to be 75 wt% by thermo-gravimetric analysis. The KO_2_-based cathode powder was prepared by high-energy planetary ball milling (Planetary Mono Mill PULVERISETTE 6 classic line, Fritsch). The mass ratio of KO_2_ (Sigma Aldrich, >99%) and RuO_2_@rGO was fixed at 6 : 4 (KO_2_: 60 wt%). The mass ratio of grinding media (zirconia ball) to material (KO_2 _+ RuO_2_@rGO) was fixed ∼8 : 1. The precursors (KO_2_-based cathode composite) were filled into the zirconia ball milling pot and sealed in the Ar-filled glove box. The rotational speed was controlled at 400 rpm for 15 min with a rest for another 3 min. The total ball milling time was around 190–200 hours. Then the as-prepared KO_2_-based cathode composite was harvested from the gas-sealed pot within the glove box.

### Cell assembly and electrochemical measurements

The electrodes were assembled into a 2032 coin cell (Hohsen Corp.). The half-cell was assembled by successively stacking a K-metal foil anode (thickness, ∼0.4 mm), the glassy fiber filter (GF/A, Whatman) with 40–45 μL of electrolyte, the Al_2_O_3_ coated polypropylene and polyethylene, and the KO_2_-based cathodic plate. For coin cell, the galvanostatic electrochemical measurements were carried out under potential control using the battery tester system HJ1001SD8 (Hokuto Denko) at 25°C. Typically, the characterizations of the cell were carried out under galvanostatic control at a current density of 300 mA g^−1^ (based on the load mass of KO_2_: 3–4 mg/cm^2^) from the open-circuit potential unless otherwise noted. For the *insitu* Raman/SERS test, the electrochemical experiments were carried out under the control of a potentiostat (Potentiostat/Galvanostat PGSTAT30, Autolab Co. Ltd., Netherlands) at room temperature. The current and potential outputs from the potentiostat were recorded by a multifunction data acquisition module/amplifier (PGSTAT30 Differential Electrometer, Autolab), which was controlled by General Purpose Electrochemical Software. Cyclic voltammetry curves were collected using the HJ1001SD8 (Hokuto Denko) system. Before each electrochemical characterization, the cells were kept on open circuit for 6–8 hours. All of the potentials in this study were referenced to K/K^+^ without further interpretation.

### Characterizations

See Supplementary Data section for details.

## Supplementary Material

nwaa287_Supplemental_File
