# Supplementary material for: A high-capacity cathode for rechargeable K-metal battery based on reversible superoxide-peroxide conversion
Source: Natl Sci Rev. 2020 Nov 27;8(10):nwaa287. doi: 10.1093/nsr/nwaa287 (PMC8566171; doi:10.1093/nsr/nwaa287)
Supplement: nwaa287_Supplemental_File [file nwaa287_supplemental_file.docx]

**Supplementary Data**

**Experimental Procedures**

**Electrolytes and Cathode Preparations**

Tetra ethylene glycol dimethylether (TEGDME, G4), 1,1,2,2-Tetrafluoroethyl 2,2,3,3-Tetrafluoropropyl ether and Bis(2,2,2-trifluoroethyl) carbonate (Sigma Aldrich, >99%) were dried over freshly activated 3 Å and 4 Å molecular sieves for several days. Potassium bis(fluorosulfonyl)imide (KFSI, purity of >98 %) and potassium bis(trifluoromethane) sulfonamide (KTFSI, purity of >98 %) salts were purchased from Tokyo Chemical Industry Co., Ltd., and dried by heating under vacuum at 80 °C oven overnight. Electrolyte was prepared and stored in a glove box under Ar atmosphere. The detailed electrolyte components: 0.5 M KTFSI, 1.0 M KFSI, 5 wt% fluorinated ether/carbonate additives. The water concentration in the electrolyte measured by Karl Fischer titration was around ~3 ppm.

For the RuO_2_@rGO based matrix, graphene Oxide (GO) was purchased from Nanjing XFNANO Materials Tech Co., Ltd, and the preparation procedure of rGO supported ultrafine RuO_2_ nanoparticles (NPs) was similar as previous reported microwave-hydrothermal synthesis method.^[^[^1^](#_ENREF_1)^]^ The RuO_2_ loading in the RuO_2_@rGO nano-composites was estimated to be 75 wt% by thermo-gravimetric analysis. The KO_2_-based cathode powder was prepared by high energy planetary ball milling (Planetary Mono Mill PULVERISETTE 6 classic line, Fritsch). The mass ratio of KO_2_ (Sigma Aldrich, >99%) and RuO_2_@rGO was fixed at 6:4 (KO_2_: 60 wt%). The mass ratio of grinding media (zirconia ball) to material (KO_2_+RuO_2_@rGO) was fixed around 8:1. The precursors (KO_2_-based cathode composite) were filled into the zirconia ball milling pot and sealed in the Ar-filled glove box. The rotational speed was controlled with 400 rpm for 15 min and rest for another 3 min. The total ball milling time was around 190~200 hours. Then the as-prepared KO_2_-based cathode composite was harvested from the gas-sealed pot within glove box.

**Cell Assembly and Electrochemical Measurements**

The electrodes were assembled into a 2032 coin cell (Hohsen Corp.). the half-cell was assembled by successively stacking a K-metal foil anode (thickness, ~0.4 mm), the glassy fiber filter (GF/A, Whatman) with 40-45 μL of electrolyte, the Al_2_O_3_ coated Polypropylene &Polyethylene (PP), and the KO_2_-based cathodic plate. For coin cell, the galvanostatic electrochemical measurements were carried out under potential control using the battery tester system HJ1001SD8 (Hokuto Denko) at 25 °C. Typically, the characterizations of the cell were carried out under galvanostatic control at a current density of 300 mA g^-1^ (based on the load mass of KO_2_­: 3~4 mg/cm^2^) from the open-circuit potential (OCP) unless other noted. For the in-situ Raman/SERS test, the electrochemical experiments were carried out under the control of a potentiostat (Potentiostat/Galvanostat PGSTAT30, Autolab Co. Ltd., Netherlands) at room temperature. The current and potential outputs from the potentiostat were recorded by a multifunction data acquisition module/amplifier (PGSTAT30 Differential Electrometer, Autolab), which was controlled by General Purpose Electrochemical Software (GPES). Cyclic voltammetry curves were collected using HJ1001SD8 (Hokuto Denko) system. Before each electrochemical characterization, the cells were kept on open circuit for 6-8 hours. All of the potentials in this study were referenced to K/K^+^ without further interpretation.

**Characterizations**

*High Resolution Transmission Electron Microscopy (HR-TEM) and X-ray Diffraction (XRD) and Measurements*

HR-TEM images were obtained using a JEM-2100 (HR) electron micro-scope. For the HR-TEM observation, the powders were ultra-sounded in dimethoxyethane (DME, Sigma Aldrich, 99%) solvent. After sufficient dispersion process, we employed a Cu mesh to harvest the dispersed particles for HR-TEM observation. X-ray diffraction measurement was performed on a Bruker D8 Advanced diffractometer fitted with Cu-Kα X-rays (λ =1.5406Å) radiation at a scan rate of 0.016 °/s. The cathode composite powder was placed into a home-made X-ray cell which was silicone glue sealed with a kapton® polyimide film. Through the measurement time of each sample was as long as 1 hour, we did not find any noticeable changes in the XRD patterns during the measurement time.

*In-situ Raman Measurements*

The in-situ Raman spectra were recorded using a JASCO microscope spectrometer (NRS-1000DT). The excitation light of an air-cooled He−Ne laser at 632.8 nm wavelength was focused on the electrode surface through a 50×long working distance lens (Olympus America Inc.). The confocal slit was adjusted to be 4.0 μm to minimize the band broadening effect due to the contribution of non-confocal signal. The scattered light was collected in a backscattering geometry along the same optical path as the pumping laser. The power of laser beam delivered to the electrode surface was roughly 10% of the maximum 30 mW laser intensity, unless specified, to avoid degradation to the products and/or carbon-based cathode. The Raman spectrum acquisition time varied from 60~90 s with 2 accumulations. At least 2 different places on the electrode surface were checked to ensure the Raman spectra were credible and reproducible. As for surface enhanced Raman spectroscopy (SERS), we took advantage of a shell-isolated nanoparticle-enhanced Raman spectroscopy (SHINERS) technique that evidently enhance the scattering signal.^[^[^2^](#_ENREF_2)^]^ Note that the amount of deposited NPs was very small, so that we assumed it would not cause any influence on the electrochemical behaviors.^[^[^3^](#_ENREF_4)^]^ The in-situ Raman cell herein was designed and modified based on the typical in-situ Raman cell (Hohsen Corp., Osaka, Japan).^[^[^4^](#_ENREF_5)^]^ In detail, a thin quartz window (thickness, 0.5 mm) has been fixed on the top of the cell as a sight window. The cathode was assembled at the bottom of the cell with the active material-face upward. On the top of the cathode, 50-100 μL of electrolyte was homogeneously dropped onto the glassy fiber filter separator (GF/A, Whatman). As a standard two-electrode configuration cell, K-metal foil (thickness, 0.4 mm) was assembled at the top as the reference and counter electrode. Note that, a small hole was punched on the center of both the separator and K-metal foil, through which the laser and Raman signals can fluidly cross.

*Gas Chromatography Mass Spectrometry (GC-MS) Characterizations*

A home-made cell for in-situ on-line differential electrochemical mass spectrometry (DEMS) and a custom-built glass vessel for ex-situ GC-MS were connected to an 8-port, two-way Gas Chromatograph (GC, Clarus@ 680, Perkin-Elmer), respectively. The inner gas circuits were modified based on those very pioneer works from B. D. McCloskey and H. A. Gasteiger.^[^[^5^](#_ENREF_7)^]^ The entire system is hermetically sealed. The Mass Spectrometer (MS, Clarus@ SQ-8 S, Perkin-Elmer) was calibrated to determine the partial pressure of standard mixture of 200 ppm O_2_ and 200 ppm CO_2_ in Ar. The estimated accuracy of gas partial pressures is approximately +10% of the measured values. The volume of the valve in mass spectrometer is 2.0 ml, and the flow velocity of the gas is controlled at 22 ml/min.

During in-situ DEMS characterization, the residual gas in the cell is flushed by Ar. Normally, the flush process would be conducted overnight (nearly 12 hours), until a stable O_2_ and CO_2_ partial pressures are obtained by monitoring the Ar pulses every 3 min. During charging, Ar gas pulses per 3 min into and outof the vessel are analyzed for O_2_ and CO_2_ partial pressure. The mass spectrometer absolute sensitivity is calibrated for CO_2_ and O_2_, therefore, the evolution rate of them can be obtained.

While For ex-situ GC-MS tests, the cathode is collected out from the cycled cell, and transfered into the glass vessel in Ar-filled golvebox without expose to open air. After the vessel is sealed and attached to the GC-MS apparatus, Ar was allowed to flow through the vessel. After obtaining a stable O_2_ and CO_2_ baselines by purging (nearly 12 hours), 1 mL of 3 M H_2_SO_4_ is injected into the vessel through a top valve Ar gas pulses per 3 min into and outof the vessel are analyzed for O_2_ and CO_2_ partial pressure until a stable O_2_ and CO_2_ baselines are obntained, and the total amount of O_2_ and CO_2_ are integrated by summing each of their contents in all pluses. Notably, trace amount of MnO_2_ powder was added into the glass vessel as the catalyst for H_2_O_2_ decompostion, and the role of MnO_2_ catalyst was essentially important for quantitative analysis, which is totally different from the titrations below.

The details of calibration processes and quantitation procedures are shown in the corresponding supporting information sections below.

(with MnO_2_, K_2_O_2_:O_2_=2:1) K_2_O_2_ + 2H_2_O = 2KOH + H_2_O +1/2O_2_

(without MnO_2_, K_2_O_2_:O_2_=n/a) K_2_O_2_ + 2H_2_O = 2KOH + H_2_O_2_

(with MnO_2_, KO_2_:O_2_=4:3) 2KO_2_ + 2H_2_O = 2KOH + H_2_O + 3/2O_2_

(without MnO_2_,KO_2_:O_2_=2:1) 2KO_2_ + 2H_2_O = 2KOH + H_2_O_2_ + O_2_

*TiOSO_4_-Based UV-Vis Spectroscopic Titrations for Peroxo- and Superoxo-Species*

The UV−vis absorption spectra were recorded using an UV−vis spectrophotometer (UV-2600, SHIMADZU) with double mono-chromators and a photomultiplier detector. A commercial UV-vis quartz cell (10 mm optical path length, JASCO Corp., Japan) was employed for both sample and reference. Before measurement, the samples were rationally diluted to avoid overranging.

The TiOSO_4_-based UV-vis titration for KO_2_ and K_2_O_2_ performed in this study have been reported in previous works.^[^[^6^](#_ENREF_13)^]^ Actually, the reaction of (hydro)peroxides with titanium (IV) and corresponding UV-vis investigations has been well studies in 1950s, and can be regarded as a powerful tool to quantitative the amount of peroxide species in the solution.^[^[^7^](#_ENREF_19)^]^ Thus, we only show a brief description of the procedures herein. The Ti^4+^-based solution was prepared with TiOSO_4_ (1g), H_2_SO_4_ (5g, 98%) and ultrapure DI-water (50 mL). Then, the harvested cathodes were directly dropped into 2.5 mL Ti^4+^-based solution without washing and evaporating procedures. The solution was vigorously shaken for 20s to promote the complete reaction of KO_2_ and K_2_O_2_ with H_2_O. The involved reaction were listed as follows:

K_2_O_2_ + 2H_2_O = 2KOH + H_2_O_2_

2KO_2_ + 2H_2_O = 2KOH + H_2_O_2_ + O_2_

(pH<3) Ti^4+^ + H_2_O_2_ + 2H_2_O = H_2_TiO_4_ (orange) + 4H^+^

(pH<3) Ti^4+^ + H_2_O_2_ = Ti(H_2_O_2_)^4+^ (orange)

(pH 3~6) Ti^4+^ + H_2_O_2_ = Ti(HO_2_)^3+^ (colorless) + H^+^

(pH 3~6) Ti^4+^ + H_2_O_2_ = Ti(O_2_)^2+^ (yellow) + 2H^+^

Then, based on the corresponding calibration curve harvested by the certain amount of hydroperoxide solution, UV-vis spectroscopy of the obtained orange (yellow) solution has been used for quantitative confirm the amount of KO_2_ and K_2_O_2_. All of the chemicals employed in titration experiments were purchased from Wako Chemical.

*Phenolphthalein-Based Acid-Base Titrations for Hydroxide*

The chemical acid-base titration processes performed in this study have been reported in related published works and our recent report.^[^[^6e^](#_ENREF_17)^,^ [^6f^](#_ENREF_18)^,^ [^8^](#_ENREF_21)^]^ The harvested cathodes were directly used without washing and evaporating procedures. Besides of this, the glass fiber separator soaked with cycled electrolyte solution was also titrated together with wet cathode in order to collect the full information of soluble products. The sample was taken out of the glove box, and put into a conical flask with 5.0 ml of ultrapure DI-water immediately. The flask was vigorously shaken for 20s to promote the complete reaction of KO_2_ and K_2_O_2_ with H_2_O:

K_2_O_2_ + 2H_2_O = 2KOH + H_2_O_2_

2KO_2_ + 2H_2_O = 2KOH + H_2_O_2_ + O_2_

In this case, the total amount of obtained base (KOH) was assign to the preloaded KO_2_ and newly-produced peroxo- and/or superoxo-species. The base was titrated using a standardized 5 mM HCl solution, with the end point indicated by 0.1 mL of phenolphthalein in isopropanol. The involved reaction is as follows:

KOH + HCl = KCl + H_2_O

*Hard X-ray photoelectron spectroscopy (XAS) measurements:*

Ex-situ Hard XAS measurements at the Ru K-edge were performed in transmission mode at the ROCK beamline15 of synchrotron SOLEIL (France). A Si(111) channel-cut quick-XAS monochromator with an energy resolution of 0.8 eV at 8 keV was used. The intensity of the monochromatic X-ray beam was measured with three consecutive ionization detectors. All data was treated using the Demeter package for energy calibration and normalization. The number of principal components was then used as the basis for multivariate curve resolution−alternating least squares (MCR−ALS) analysis. Finally, the ex-situ spectra were analysed by using Artemis software. The RuO_2_ for XAS was prepared as a standard reference.^[^[^9^](#_ENREF_25)^]^

**Supplementary Discussion**

**Thermodynamic potentials/capacities for typical redox reactions:**

Δ_f_G^0^ (K_2_O) = -321.17 kJ/mol; (94.2 g/mol)

Δ_f_G^0^ (K_2_O_2_) = -425.1 kJ/mol; (110.2 g/mol)

Δ_f_G^0^ (KO_2_) = -239.41 kJ/mol; (71.1 g/mol)

(1 kcal/mol = 4.184 kJ/mol; 1eV = 23.06 kcal/mol)

According to the isothermal relation of Gibbs free energy and electromotive force: ΔG = -nFE; ΔG stands for the change of the Gibbs free energy during a reaction; n stands for the electron number transferred in the reaction; F stands for Faradays Constant: 96485.33 C mol^−1^; E stands for the electromotive force of the reaction. The related gravimetric specific capacity are calculated based on corresponding discharge products. The thermodynamic parameters are collected based on classic reported papers.^[^[^10^](#_ENREF_26)^]^

The thermodynamic oxygen evolution (OER) potential:

O_2_ + K^+^ + e^-^ = KO_2_; E^0^ = 2.48 V vs. K/K^+^;

O_2_ + 2K^+^ + 2e^-^ = K_2_O_2_; E^0^ = 2.2 V vs. K/K^+^;

O_2_ + 4K^+^ + 4e^-^ = 2K_2_O; E^0^ = 1.66 V vs. K/K^+^;

Oxygen-related redox couple conversion reactions (without O_2_ evolution):

KO_2_ + K^+^ + e^-^ = K_2_O_2_; E^0^ = 1.92 V vs. K/K^+^; (377 mAh/g_~KO2_)

KO_2_ + 3K^+^ + 3e^-^ = 2K_2_O; E^0^ = 1.39 V vs. K/K^+^;

K_2_O_2_ + 2K^+^ + 2e^-^ = 2K_2_O; E^0^ = 1.125 V vs. K/K^+^;

**Supplementary Figures**

**Table S1.** Parameters summary of electrode (cathode/anode) materials studied for their application in KIBs represented by their specific capacity; operating potential; average potential plateau, etc.^[^[^11^](#_ENREF_31)^]^


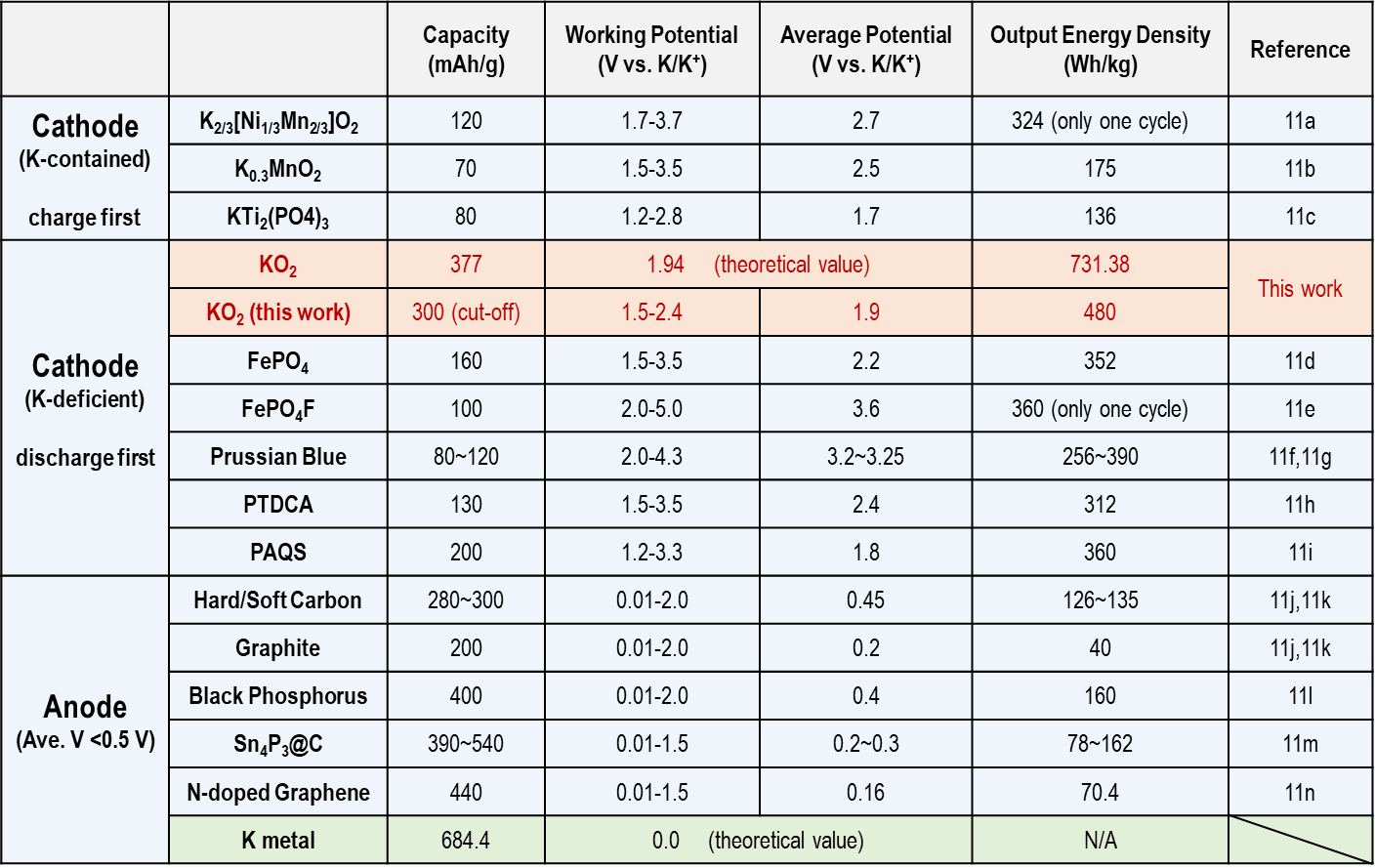


**
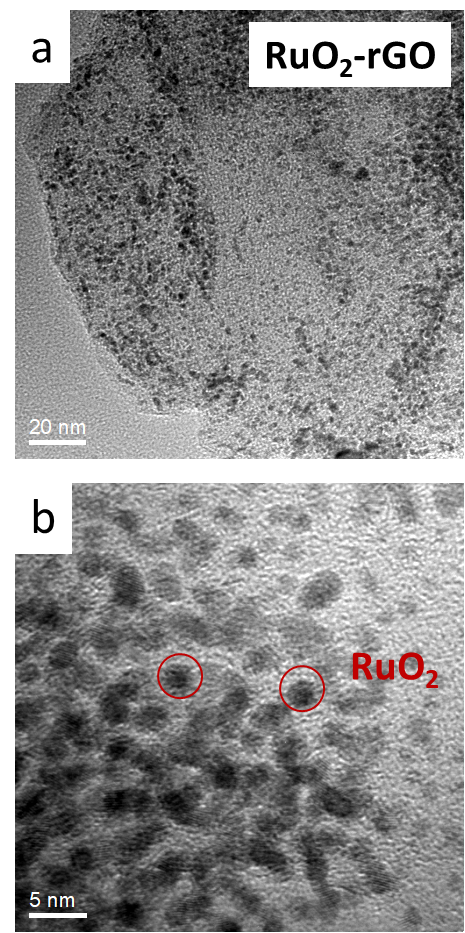
Figure S1.** (a-b) HR-TEM images of the RuO_2_@rGO matrix. The images are collected based on dispersed mono-layer 2D rGO sheets.


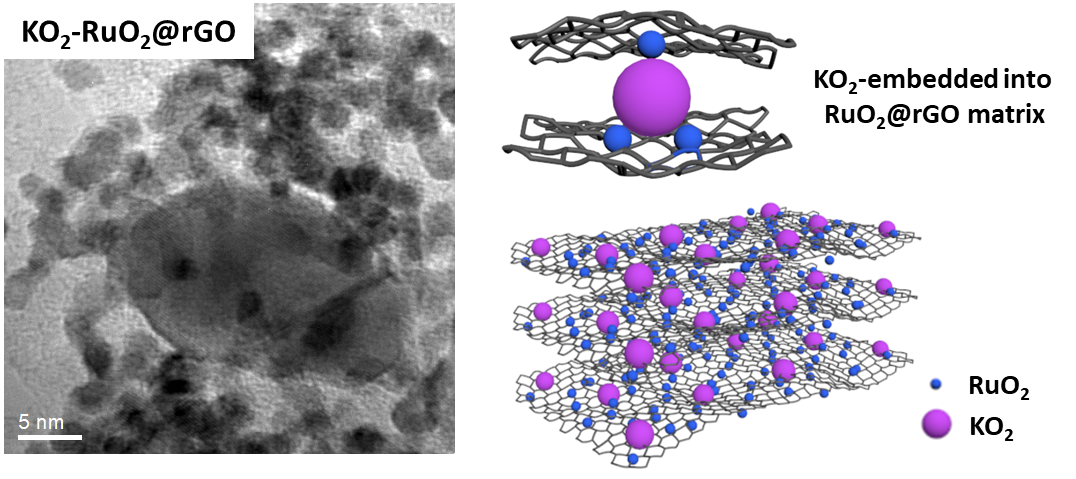
**Figure S2.** Zoom-in HR-TEM image of the KO_2_-embedded RuO_2_@rGO. The corresponding schematic diagram is shown inset.


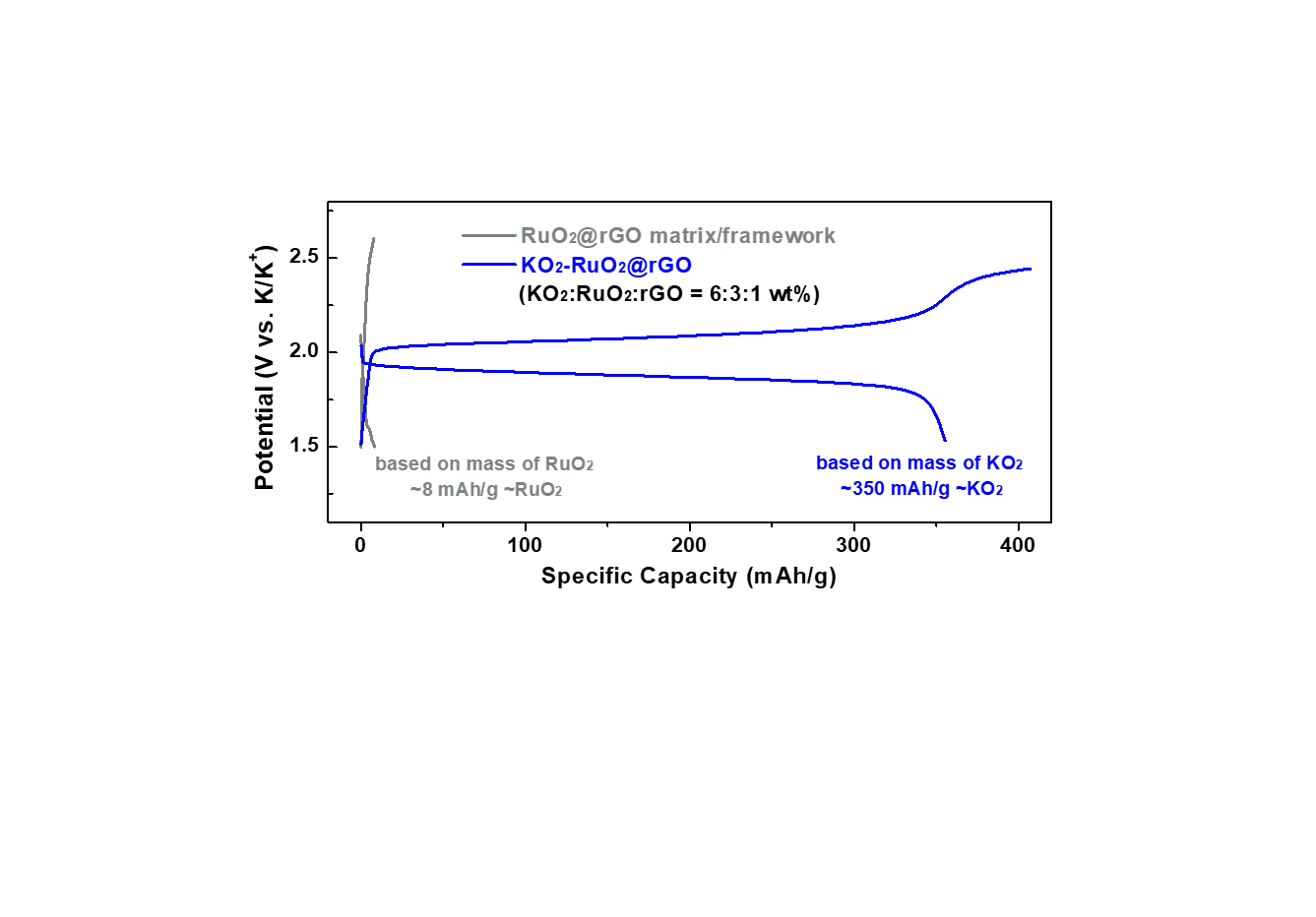
**Figure S3.** Galvanostatic discharge/charge curves of RuO_2_@rGO substrate. Specific capacity is based on mass of RuO_2_ and pre-loaded KO_2_, respectively. The RuO_2_@rGO substrate has been demonstrated to have negligible contribution to cell capacity.


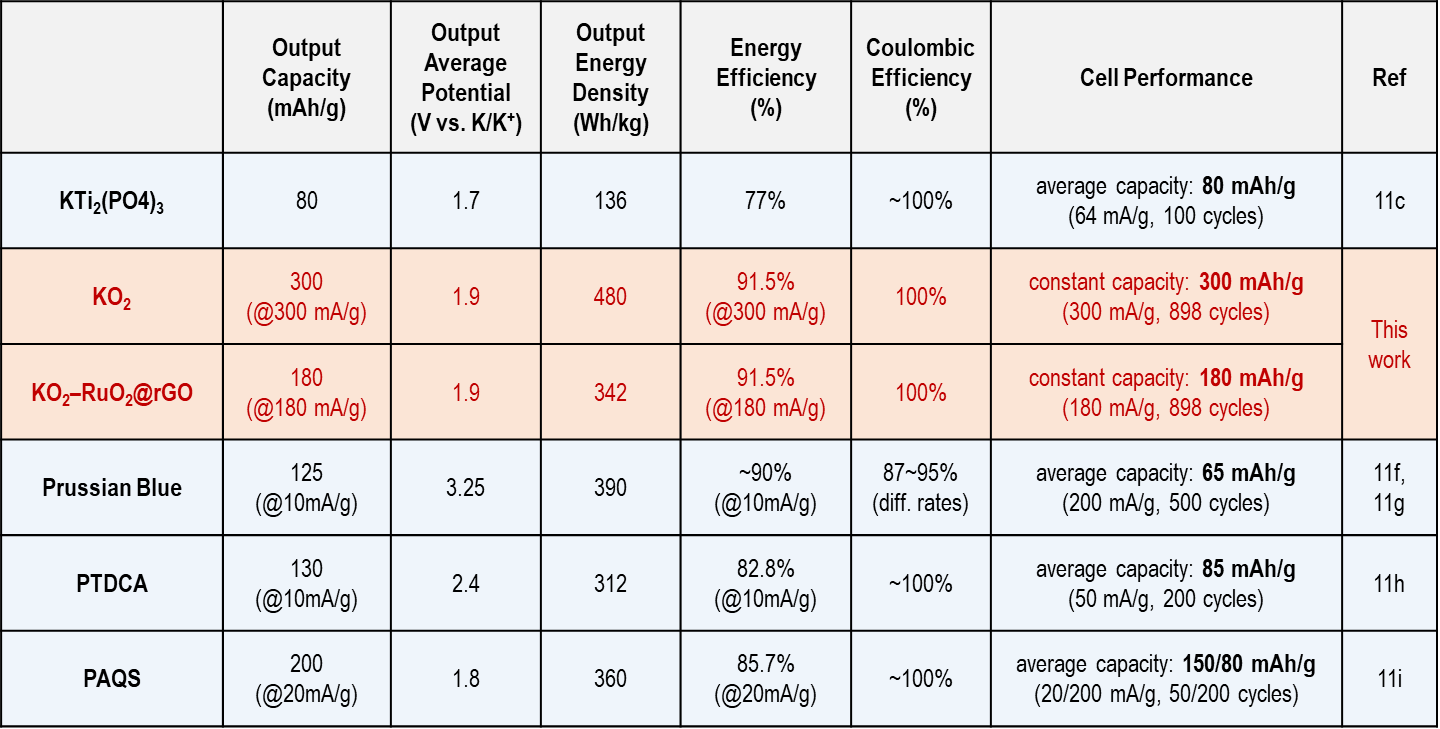
**Table S2.** Summary of half-cell cycling performance of various cathode candidates, on the aspects of output capacity, round-trip efficiency, coulombic efficiency and corresponding cycling performance.^[^[^11c^](#_ENREF_33)^,^ [^11f-i^](#_ENREF_36)^]^ Additionally, the mass loading of KO_2_ is fixed around 3~4 mg/cm^2^ (KO_2_-RuO_2_@rGO: 5~6.5 mg/cm^2^).


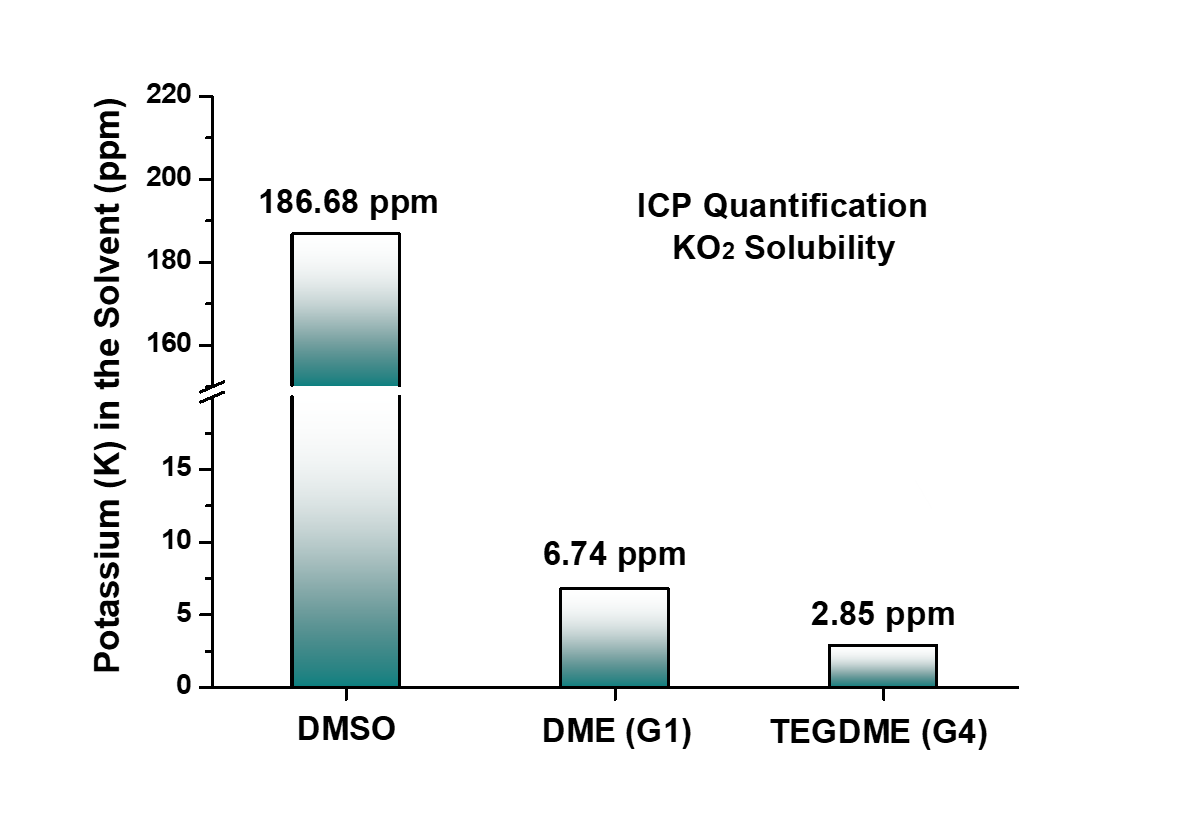
**Figure S4.** ICP-MS characterization for the solubility of KO_2_ in related electrolyte solvent: DMSO, DME and TEGDME, respectively.


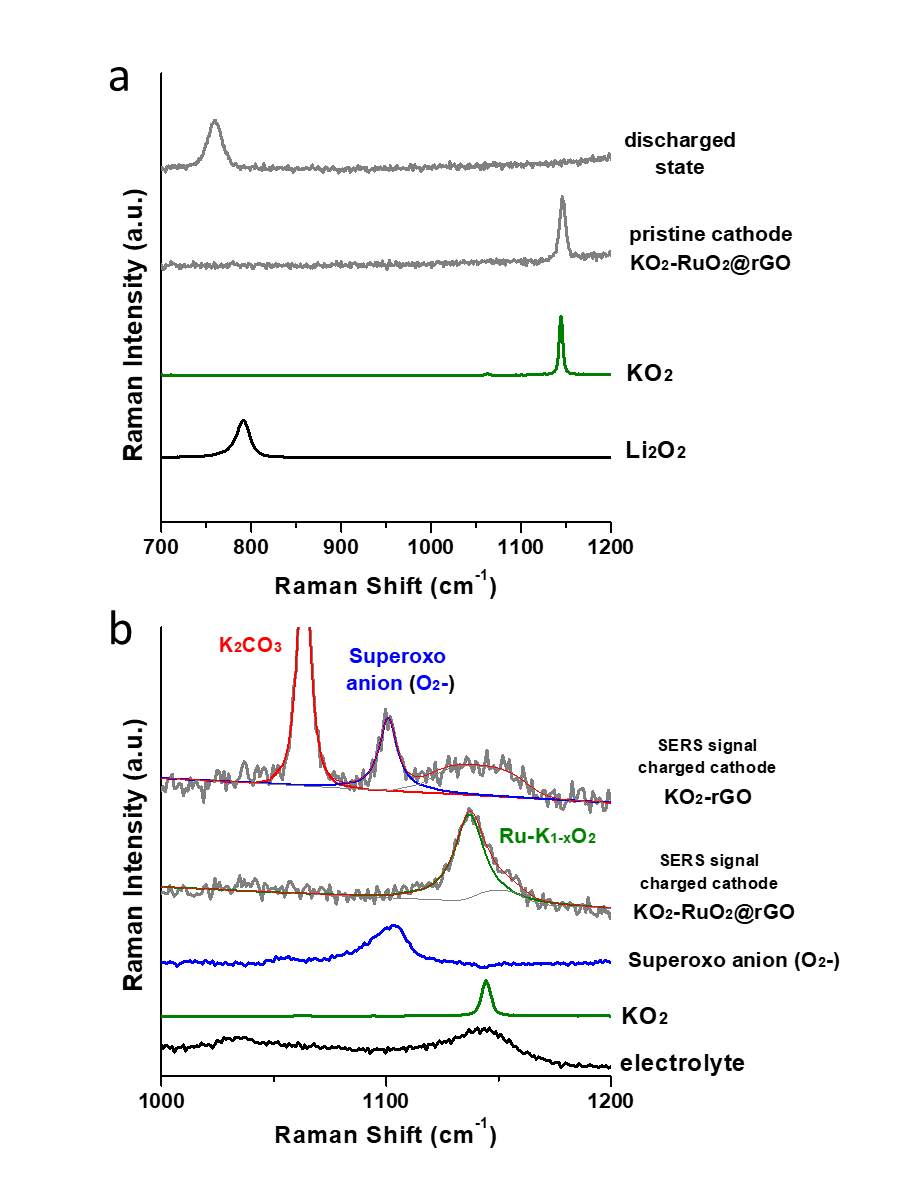
**Figure S5.** Assignments of (a) Typical Raman spectra and (b) Surface-enhanced Raman (SERS) signals. The corresponding standard/reference spectra are shown for comparison. As for the spectrum of adsorbed superoxide anion (O_2_^-^), the SERS signal (differential/subtraction spectrum) is collected from a gold cathode during ORR in TBAClO_4_-DMSO electrolyte in previous study.^[^[^12^](#_ENREF_45)^]^


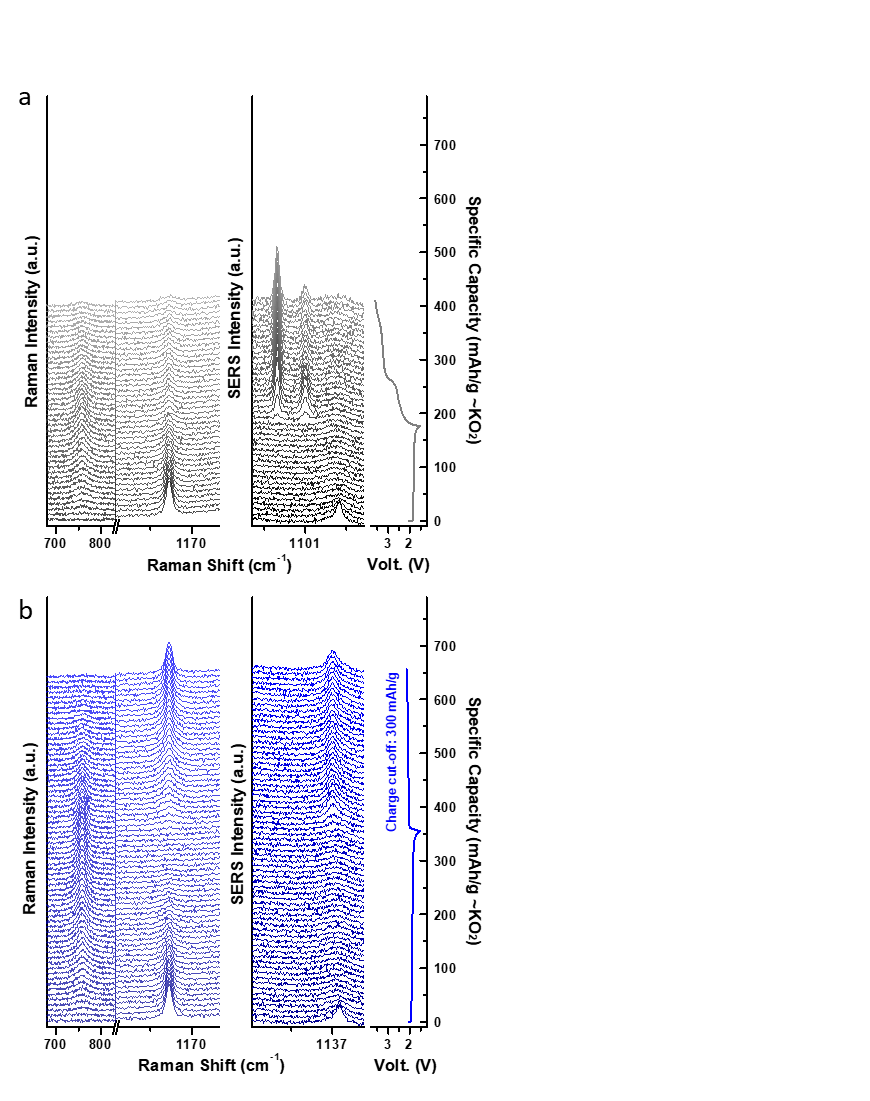
**Figure S6.** (a) Typical bulk-sensitive operando Raman spectra and (b) Surface-sensitive surface-enhanced operando SHINERS spectra observed on KO_2_-rGO and KO_2_-RuO_2_@rGO cathodes recorded during initial galvanostatic cycle. The capacity-dependent spectra are offset without overlap with related spectral contour plots (as shown in the manuscript, Figure 2b and 2c).


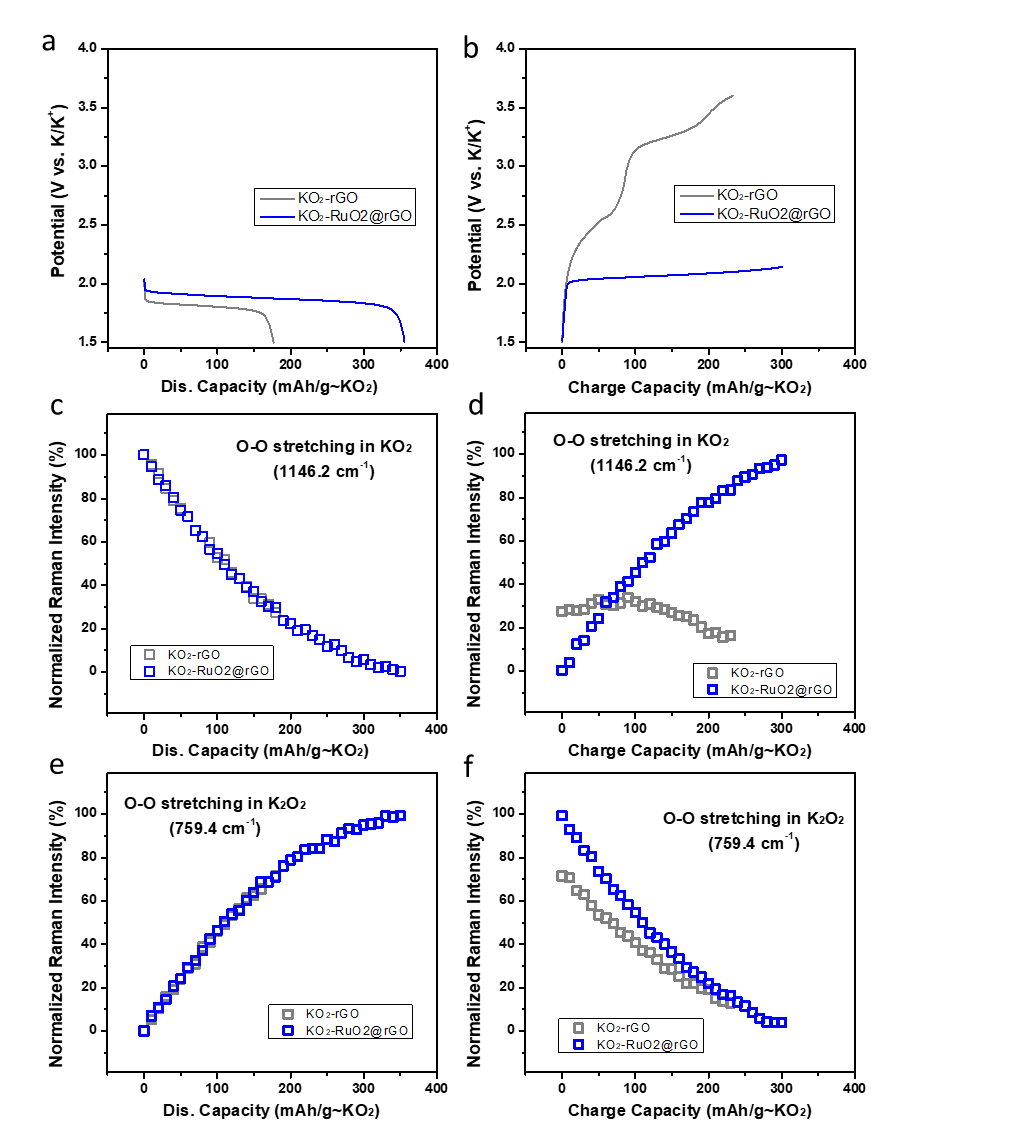
**Figure S7.** Capacity dependence of two Raman peaks (O-O stretching modes in KO_2_ and K_2_O_2_, respectively) observed on the KO_2_-based cathodes (KO_2_-rGO and KO_2_-RuO_2_@rGO, respectively). The peak intensities are directly read from the in situ Raman spectra.


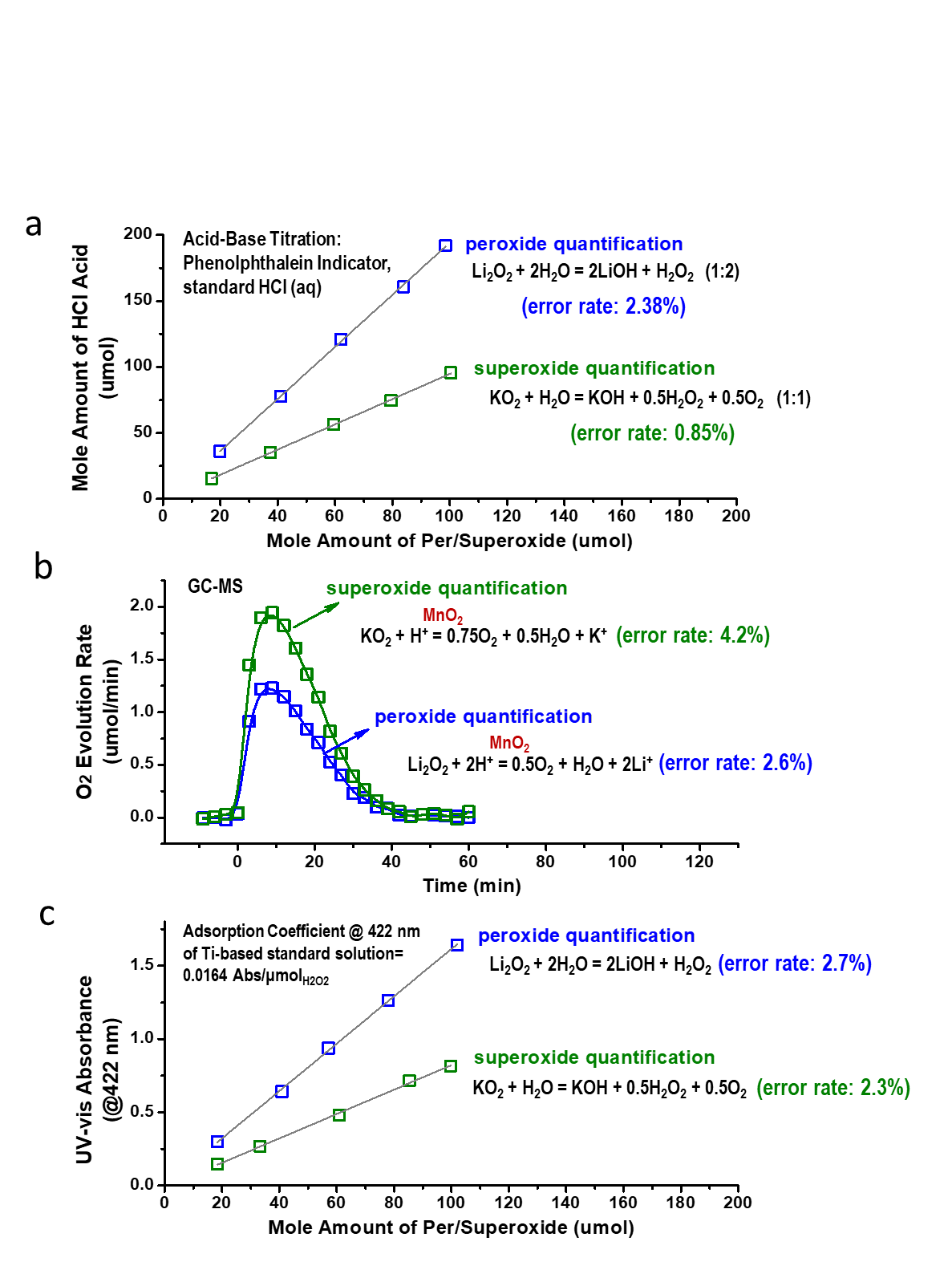
**Figure S8.** The currently-introduced “double-check” quantification method for KO_2_ and K_2_O_2_ products: the combination of titrations (phenolphthalein-based acid-base titration and TiOSO_4_-Based UV-vis spectroscopic Titrations) and a gas chromatograph-mass spectrometer (GC-MS) characterization.

The principle of “double-check” quantification method: Cathode deposited species: x mol KO_2_ and y mol K_2_O_2_.

(1) Phenolphthalein-based acid-base titration (HCl consumption: a mol):

K_2_O_2_ + 2H_2_O = 2KOH + H_2_O_2_

2KO_2_ + 2H_2_O = 2KOH + H_2_O_2_ + O_2_

KOH + HCl = KCl + H_2_O

Eq 1. x + 2y = a

(2) TiOSO_4_-Based UV-vis spectroscopic Titrations (Ti(O_2_)^2+^: b mol):

K_2_O_2_ + 2H_2_O = 2KOH + H_2_O_2_

2KO_2_ + 2H_2_O = 2KOH + H_2_O_2_ + O_2_

(pH 3~6) Ti^4+^ + H_2_O_2_ = Ti(O_2_)^2+^ (yellow) + 2H^+^

Eq 2. x + 2y = 2b

(3) GC-MS characterization (O_2_ evolution: c mol):

K_2_O_2_ + 2H_2_O = 2KOH + H_2_O +1/2O_2_ (K_2_O_2_:O_2_=2:1)

2KO_2_ + 2H_2_O = 2KOH + H_2_O + 3/2O_2_ (KO_2_:O_2_=4:3)

Eq 3. 3x + y = 4c

Notably, there is different between Phenolphthalein-based acid-base titration (2KO_2_ + 2H_2_O = 2KOH + H_2_O_2_ + O_2_) and GC-MS characterization (2KO_2_ + 2H_2_O = 2KOH + H_2_O + 3/2O_2_), which is ascribed to the MnO_2_ catalyst (towards OER of H_2_O_2_) has been provided in GC-MS characterization.

In this case, the eq.1 and eq.3 compose the 1^st^ round check equation set:

x + 2y = a; 3x + y = 4c

While, the eq.2 and eq.3 compose the 2^nd^ round check equation set:

x + 2y = 2b; 3x + y = 4c

**Figure S9.** Quantification results (towards KO_2_ and K_2_O_2_) harvested based on the “double-check” quantification characterizations. Experimental data collection points: pristine state (grey blocks); discharge/charge states of the 1^st^ cycle (blue blocks) and the 5^th^ cycle (green blocks). The error bar is obtained by the error quantified by “double-check” equation sets. The theoretical values (calculation based on practical capacity) are highlighted by the red trace and dash line.


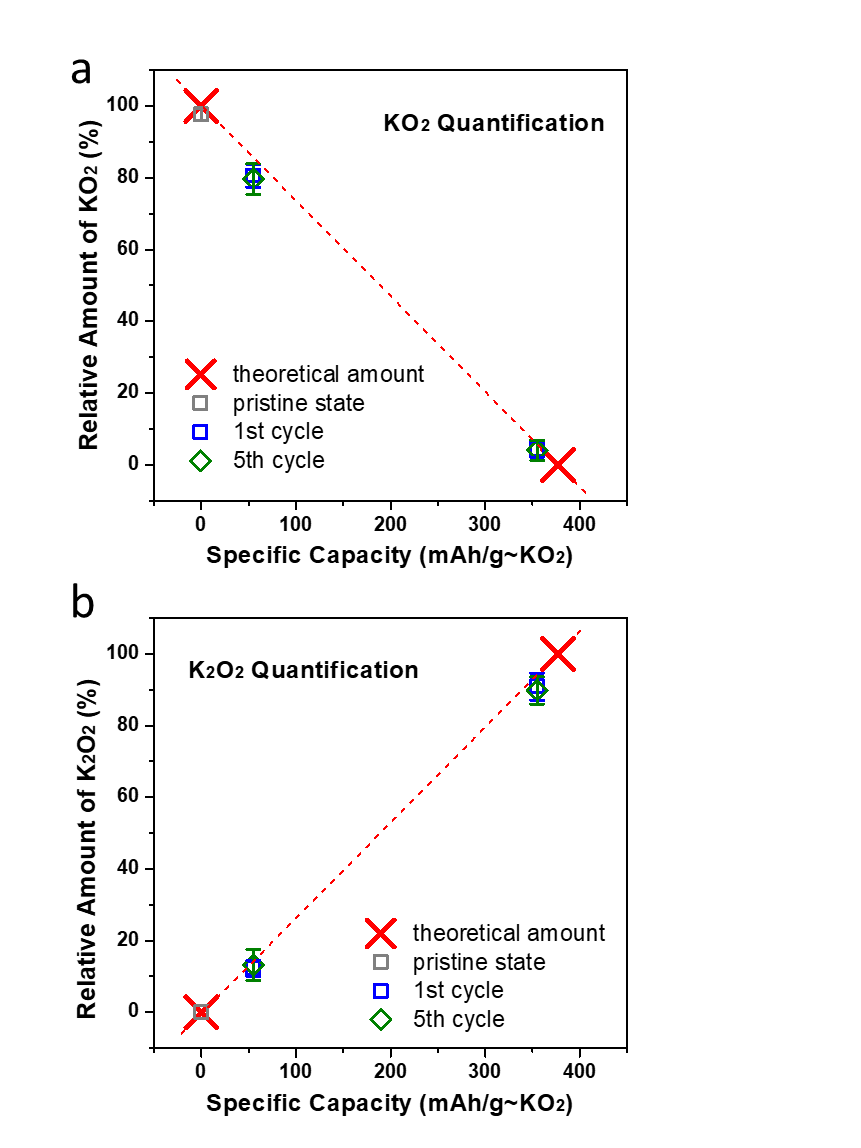


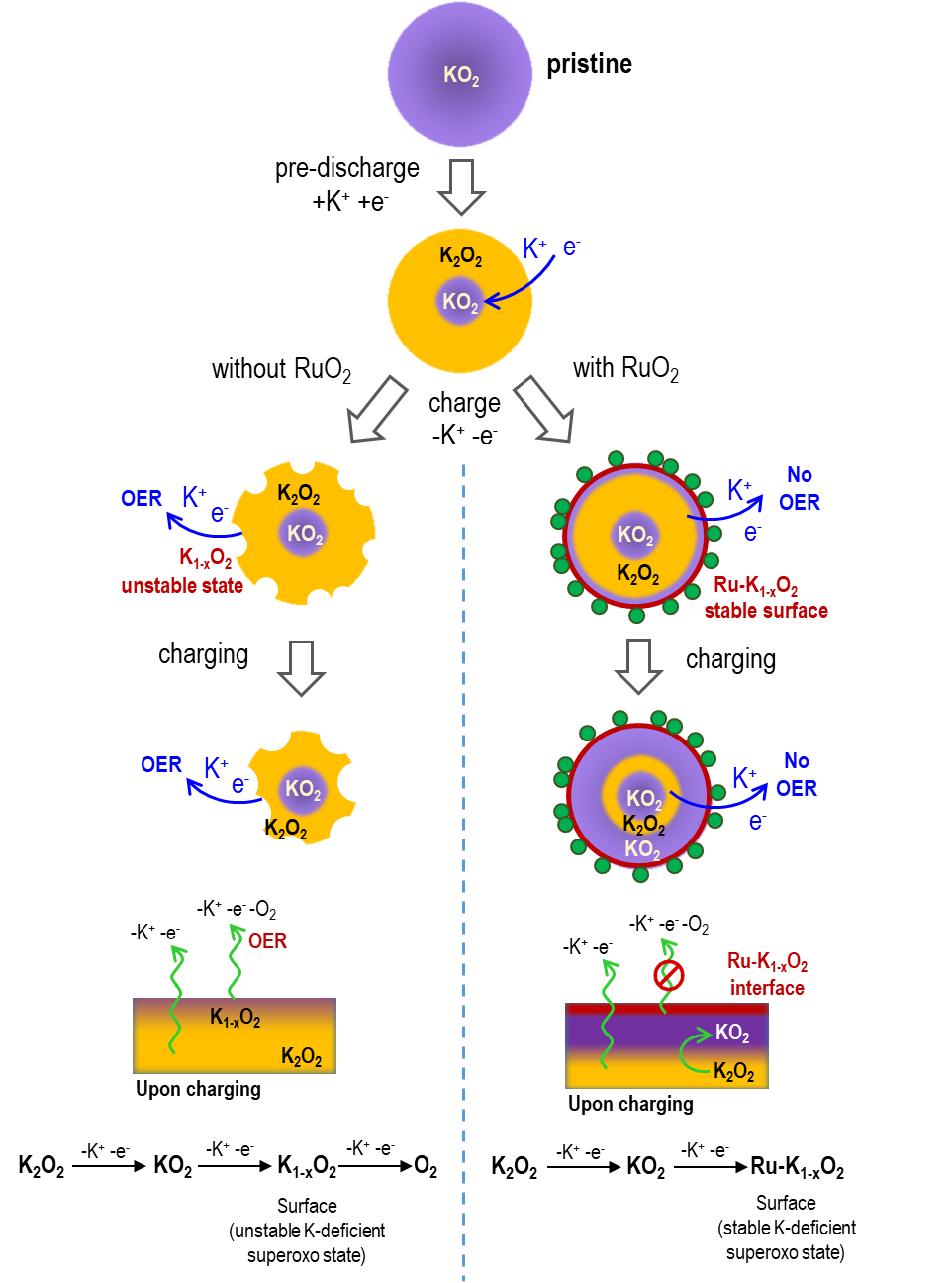
**Figure S10.** Schematic of the reaction (electrochemical redox) pathway on KO_2_-based cathodes (KO_2_-rGO and KO_2_-RuO_2_@rGO, respectively).


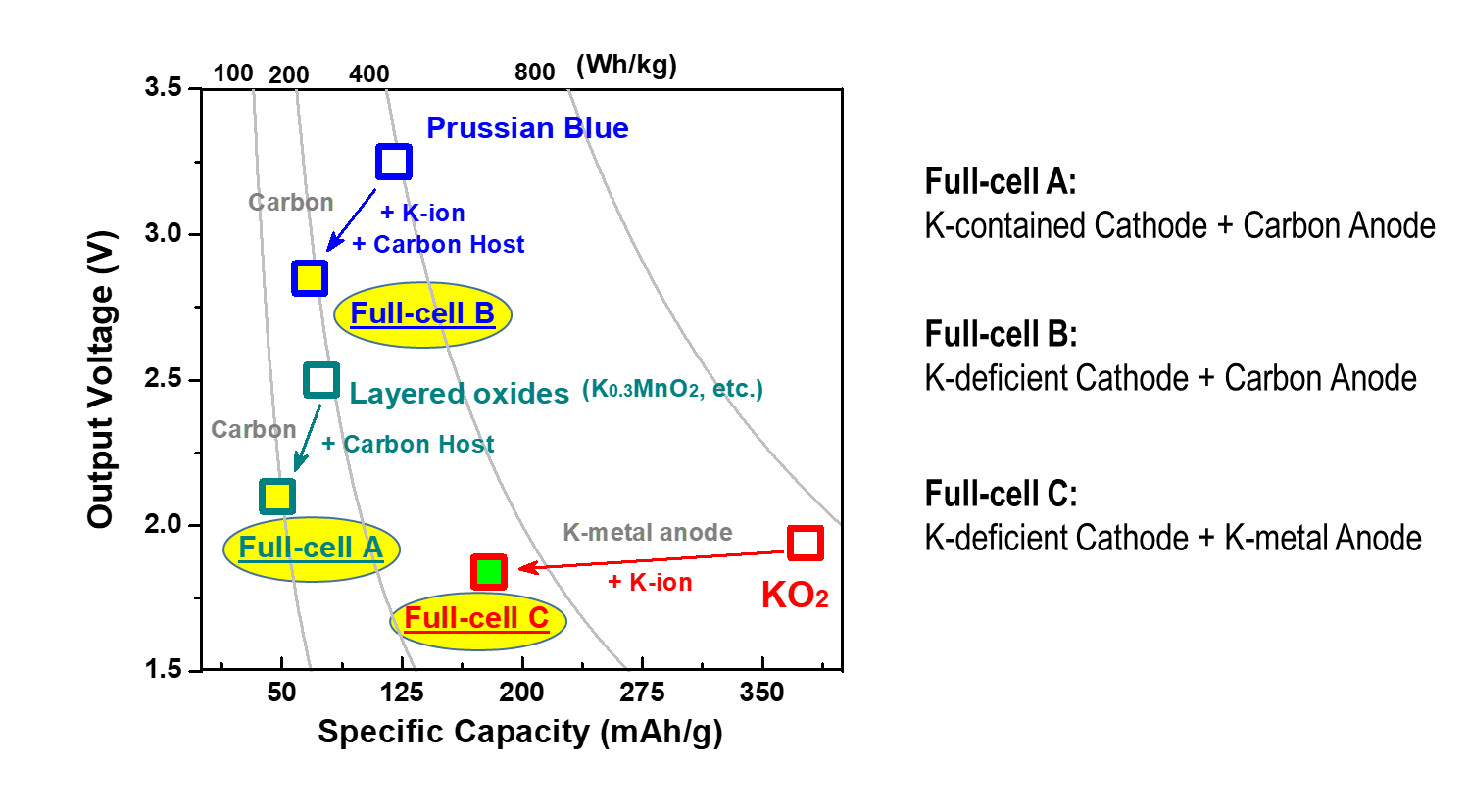
**Figure S11.** Calculation of full-cell out-put energy density (based on the combination of different cathode states and anode candidates, respectively).

**General principle:**

**Full-cell output voltage:** V_full-cell_ = V_c_ – V_a_;

V_c_/V_a_: average discharge/charge potential of cathode/anode in corresponding half-cell.

**Full-cell output capacity**: Cap_full-cell_ = Q / (M_c_ + M_a_ + η);

Q = 26760 mAh (electric quantity of 1 mol electron); M_c_/M_a_: the mole mass of cathode/anode; η is a specific parameter: for K-contained cathode, η = 0; as for a K-deficient cathode, the mass of additional of K-ion must be fairly taken into consideration η = M_potassium_ = 39.1 g/mol.

**A) Full-cell A:** Layered oxide cathode (K-contained) and carbon anode:

Cap_full-cell_ = 46.05 mAh/g

(M_c_ = 356 g/mol, M_carbon_ = (26760 mAh) / (200 mAh/g) = 133.8 g/mol)

**B) Full-cell B:** Prussian blue cathode (K-deficient) and carbon anode:

Cap_full-cell_ = 67.74 mAh/g

(M_c_ = (26760 mAh) / (120 mAh/g) = 223 g/mol, M_carbon_ = 133.8 g/mol)

Actually, at specific highly-concentrated electrolyte system, the specific capacity of graphite can reaches ~250 mAh/g.^[^[^13^](#_ENREF_46)^]^ Herein, we employ a typical specific capcity value (~200 mAh/g) for graphite anode.

**C) Full-cell C:** KO_2_­ cathode (K-deficient) and Cu (No anode):

Cap_full-cell_ = 242.83 mAh/g

(Mc = 71.1 g/mol, η = M_potassium_ = 39.1 g/mol)

**D) Full-cell D:** KO_2_­ cathode (K-deficient) and K-metal anode (100% excess amount):

Cap_full-cell_ = 179.2 mAh/g

(Mc = 71.1 g/mol, M_K-metal_ = 39.1 g/mol, η = M_potassium_ = 39.1 g/mol)


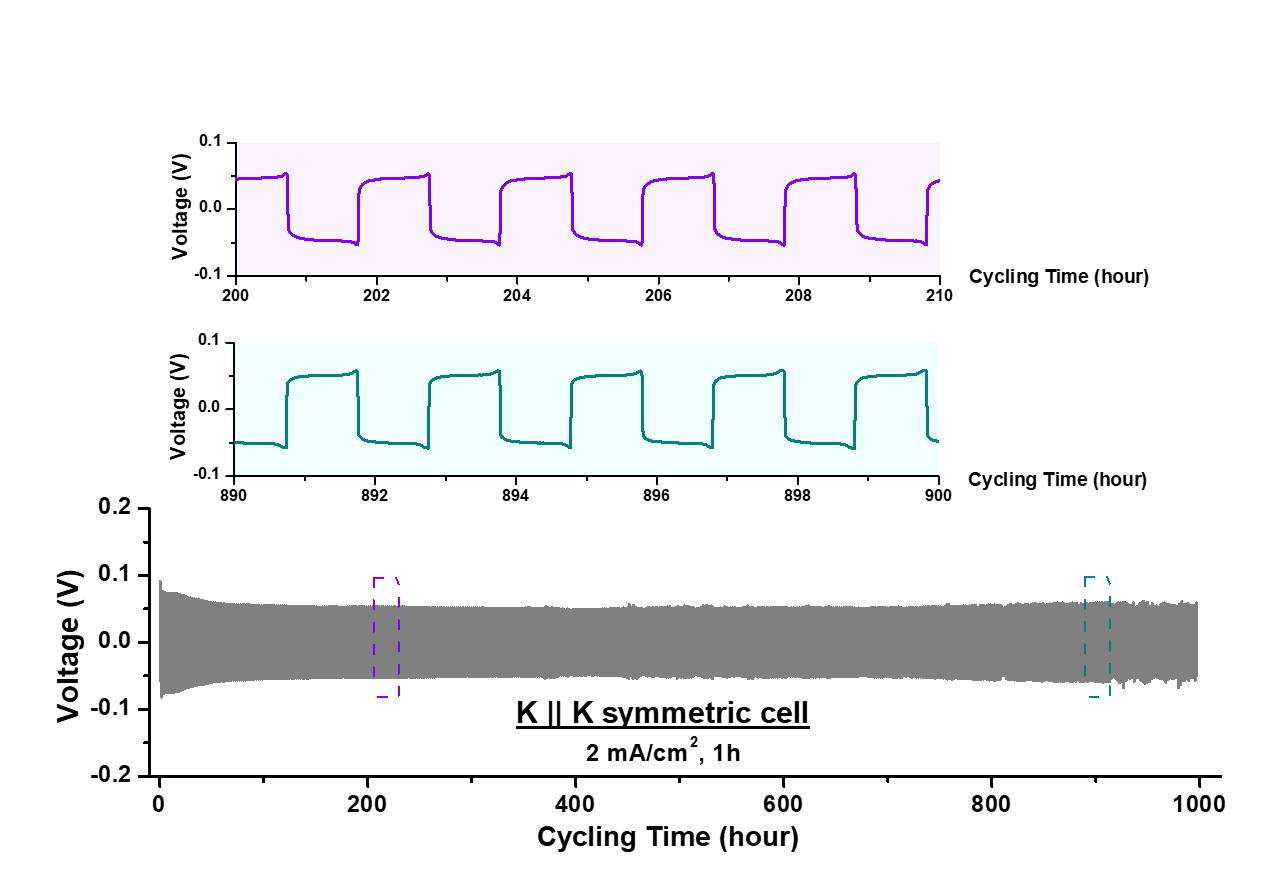
**Figure S12.** Performance of K|| K symmetric cell. Current density: at 2 mA/cm^2^, for 1 hour. The electrolyte system: 0.5 M KTFSI + 1.0 M KFSI (dual salts) in TEGDME with 5 wt% 1,1,2,2-Tetrafluoroethyl 2,2,3,3-Tetrafluoropropyl ether (fluorinated ether). The typical curves are shown inset for clarity.


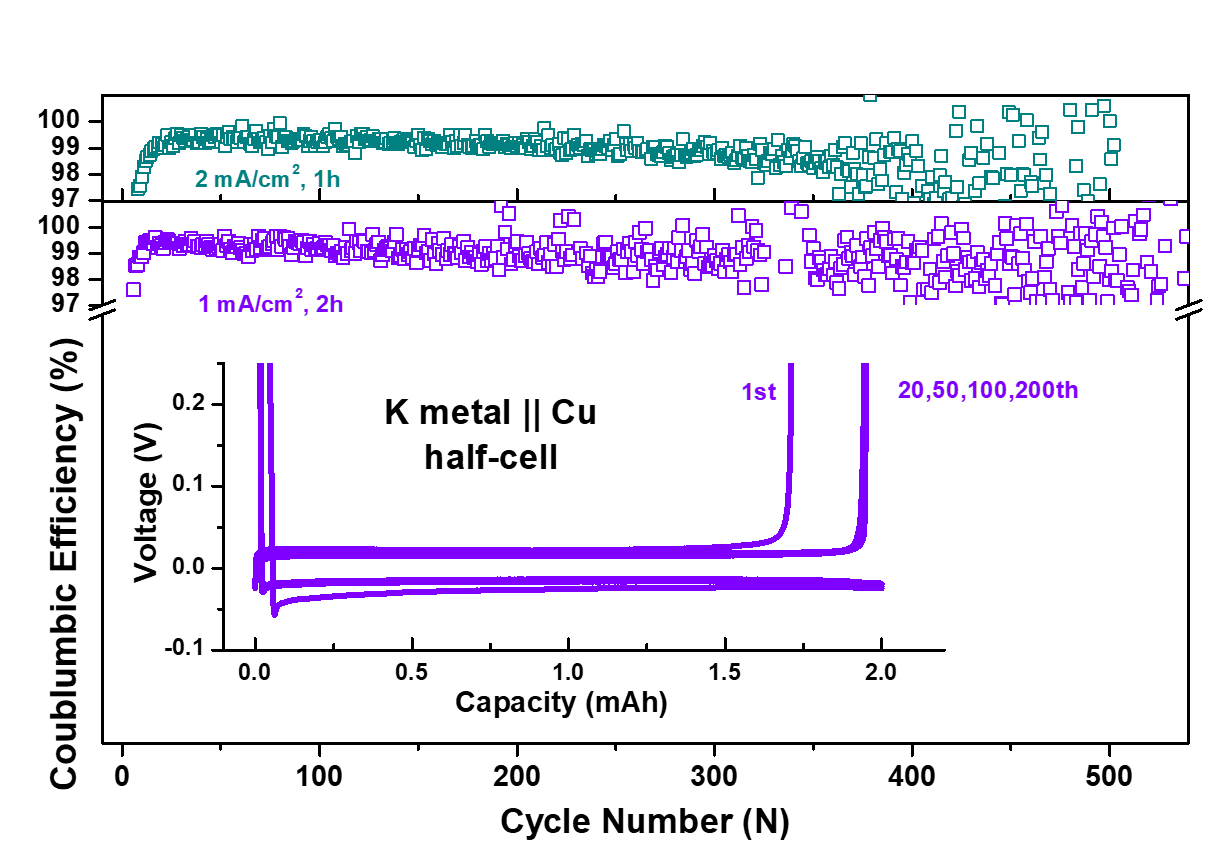
**Figure S13.** Cycle stability (Coulombic Efficiency, CE%) of K || Cu half-cell. The electrolyte system: 0.5 M KTFSI + 1.0 M KFSI (dual salts) in TEGDME with 5 wt% 1,1,2,2-Tetrafluoroethyl 2,2,3,3-Tetrafluoropropyl ether (fluorinated ether). The typical curves are shown inset for clarity.


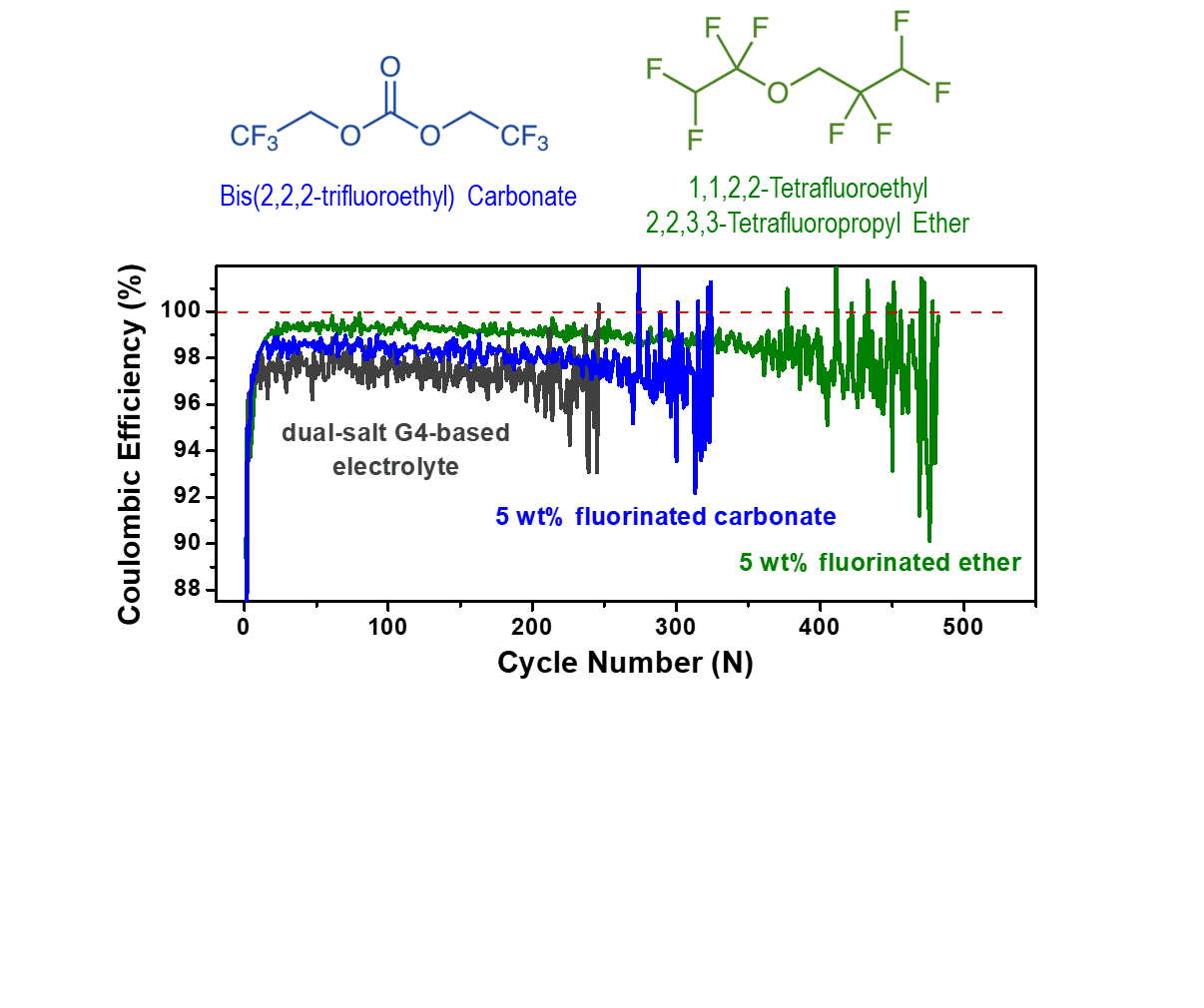
**Figure S14.** Cycle stability (Coulombic Efficiency, CE%) of K || Cu half-cell assembled with different fluorinated additive. Basic electrolyte system: dual-salts (0.5 M KTFSI + 1.0 M KFSI) TEGDME. Fluorinated additives (5 wt%): 2,2,3,3-Tetrafluoropropyl ether (green trace); Bis(2,2,2-trifluoroethyl) carbonate (blue trace).


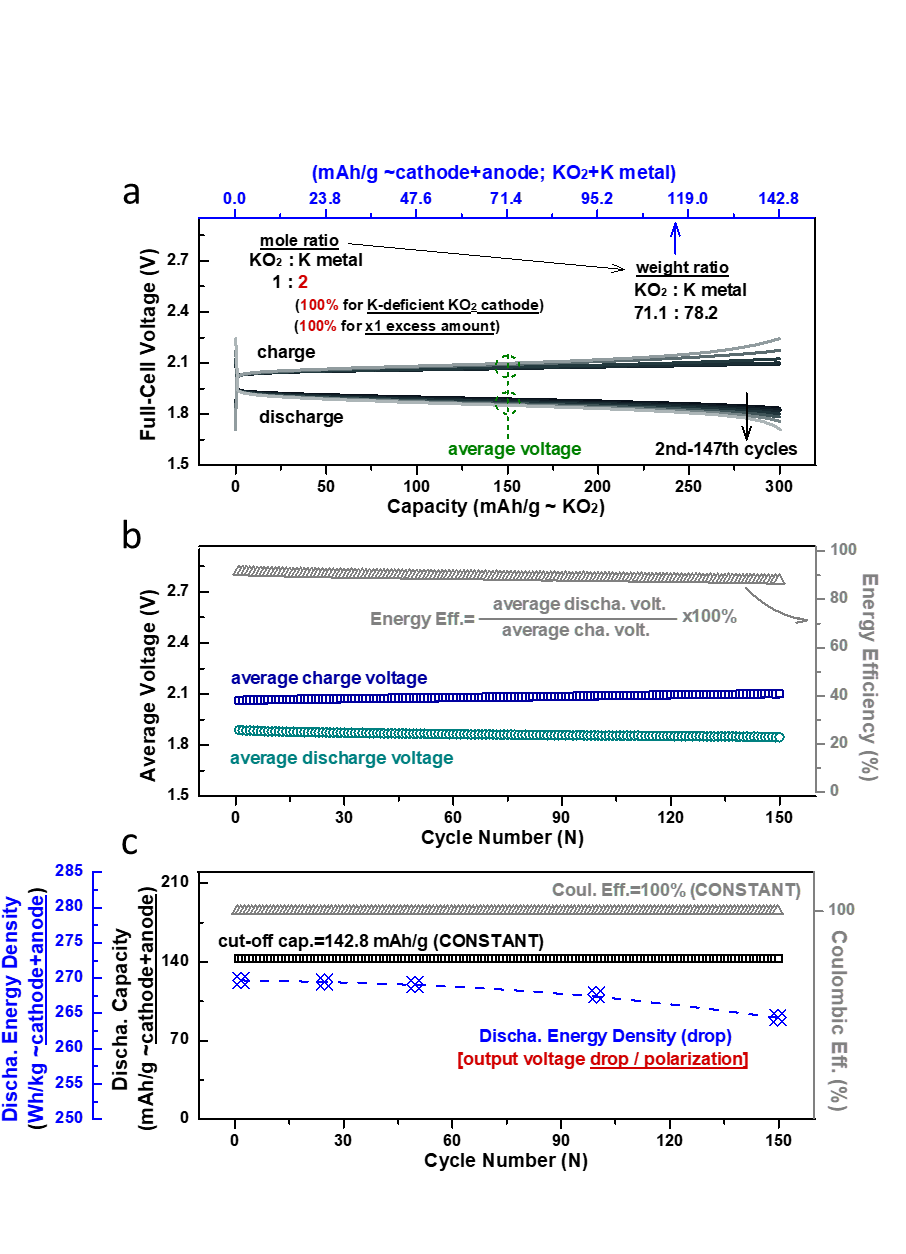
**Figure S15.** Summary of parameters within K-metal full-cell. Cathode: KO_2_-based cathode with 300 mAh/g~KO_2_ cut-off capacity; Anode: 100% excess amount of K-metal. Notably, as shown in Figure a, since the KO2-based presents a K-deficient state, thus, the K-ion should be provided for the 1^st^ pre-discharged process. During energy density calculation, we fairly take this mass of K-metal into consideration.

**Supplemental References**

[1] J.-Y. Kim, K.-H. Kim, S.-B. Yoon, H.-K. Kim, S.-H. Park, K.-B. Kim, *Nanoscale* **2013**, *5*, 6804-6811.

[2] a) G. Frens, *Nat. Phys. Sci.* **1973**, *241*, 20-22; b) J. F. Li, Y. F. Huang, Y. Ding, Z. L. Yang, S. B. Li, X. S. Zhou, F. R. Fan, W. Zhang, Z. Y. Zhou, D. Y. Wu, B. Ren, Z. L. Wang, Z. Q. Tian, *Nature* **2010**, *464*, 392-395.

[3] S. Hy, F. Felix, J. Rick, W.-N. Su, B. J. Hwang, *J. Am. Chem. Soc.* **2014**, *136*, 999-1007.

[4] a) Y. Qiao, J. Yi, S. Wu, Y. Liu, S. Yang, P. He, H. Zhou, *Joule* **2017**, *1*, 359-370; b) Y. Qiao, Y. He, S. Wu, K. Jiang, X. Li, S. Guo, P. He, H. Zhou, *ACS Energy Lett.* **2018**, *3*, 463-468.

[5] a) B. D. McCloskey, D. S. Bethune, R. M. Shelby, G. Girishkumar, A. C. Luntz, *J. Phys. Chem. Lett.* **2011**, *2*, 1161-1166; b) B. D. McCloskey, D. S. Bethune, R. M. Shelby, T. Mori, R. Scheffler, A. Speidel, M. Sherwood, A. C. Luntz, *J. Phys. Chem. Lett.* **2012**, *3*, 3043-3047; c) B. D. McCloskey, R. Scheffler, A. Speidel, G. Girishkumar, A. C. Luntz, *J. Phys. Chem. C* **2012**, *116*, 23897-23905; d) S. Meini, S. Solchenbach, M. Piana, H. A. Gasteiger, *J Electrochem Soc* **2014**, *161*, A1306-A1314; e) S. Meini, M. Piana, N. Tsiouvaras, A. Garsuch, H. A. Gasteiger, *Electrochem Solid St* **2012**, *15*, A45-A48; f) H. Beyer, M. Metzger, J. Sicklinger, X. Wu, K. U. Schwenke, H. A. Gasteiger, *J. Electrochem. Soc.* **2017**, *164*, A1026-A1036.

[6] a) K. U. Schwenke, M. Metzger, T. Restle, M. Piana, H. A. Gasteiger, *J. Electrochem. Soc.* **2015**, *162*, A573-A584; b) W.-J. Kwak, S. H. Ha, D. H. Kim, K. H. Shin, Y.-K. Sun, Y. J. Lee, *ACS Catal.* **2017**, 8192-8199; c) B. Schafzahl, E. Mourad, L. Schafzahl, Y. K. Petit, A. R. Raju, M. O. Thotiyl, M. Wilkening, C. Slugovc, S. A. Freunberger, *ACS Energy Lett.* **2018**, *3*, 170-176; d) N. Xiao, R. T. Rooney, A. A. Gewirth, Y. Wu, *Angew. Chem. Int. Ed.* **2018**, n/a-n/a; e) Y. Qiao, K. Jiang, H. Deng, H. Zhou, *Nat Catalysis* **2019**, *2*, 1035-1044; f) Y. Qiao, Q. Wang, X. Mu, H. Deng, P. He, J. Yu, H. Zhou, *Joule* **2019**, *3*, 2986 - 3001.

[7] a) G. Eisenberg, *Industrial & Engineering Chemistry Analytical Edition* **1943**, *15*, 327-328; b) C. N. Satterfield, A. H. Bonnell, *Anal. Chem.* **1955**, *27*, 1174-1175.

[8] a) Y. Qiao, S. Wu, J. Yi, Y. Sun, S. Guo, S. Yang, P. He, H. Zhou, *Angew. Chem. Int. Ed.* **2017**, *56*, 4960-4964; b) F. J. Li, S. C. Wu, D. Li, T. Zhang, P. He, A. Yamada, H. S. Zhou, *Nat. Common.* **2015**, *6*, 8843; c) N. B. Aetukuri, B. D. McCloskey, J. M. Garcia, L. E. Krupp, V. Viswanathan, A. C. Luntz, *Nat. Chem.* **2015**, *7*, 50-56; d) C. Xia, C. Y. Kwok, L. F. Nazar, *Science* **2018**, *361*, 777.

[9] X. Cao, H. Li, Y. Qiao, X. Li, M. Jia, J. Cabana, H. Zhou, *Adv Energy Mater* **2020**, *n/a*, 1903785.

[10] a) Y. N. Zhuravlev, Y. M. Basalaev, A. S. Poplavnoi, *Theoretical and Experimental Chemistry* **2003**, *39*, 81-84; b) D. V. Zhdanov, M. A. Ul’yanova, Y. A. Ferapontov, *Russian Journal of Applied Chemistry* **2005**, *78*, 184-187; c) A. K. Nandy, P. Mahadevan, D. D. Sarma, *Phys. Rev. B* **2011**, *84*, 035116; d) A. K. Nandy, P. Mahadevan, D. D. Sarma, *Molecular Simulation* **2012**, *38*, 1308-1314; e) J. Yan, J. S. Hummelshøj, J. K. Nørskov, *Phys. Rev. B* **2013**, *87*, 075207.

[11] a) M. G. T. Nathan, N. Naveen, W. B. Park, K.-S. Sohn, M. Pyo, *Journal of Power Sources* **2019**, *438*, 226992; b) J. C. Pramudita, D. Sehrawat, D. Goonetilleke, N. Sharma, *Adv. Energy Mater.* **2017**, *7*, 1602911; c) J. Han, Y. Niu, S.-j. Bao, Y.-N. Yu, S.-Y. Lu, M. Xu, *Chem Commun* **2016**, *52*, 11661-11664; d) V. Mathew, S. Kim, J. Kang, J. Gim, J. Song, J. P. Baboo, W. Park, D. Ahn, J. Han, L. Gu, Y. Wang, Y.-S. Hu, Y.-K. Sun, J. Kim, *NPG Asia Materials* **2014**, *6*, e138-e138; e) N. Recham, G. Rousse, M. T. Sougrati, J.-N. Chotard, C. Frayret, S. Mariyappan, B. C. Melot, J.-C. Jumas, J.-M. Tarascon, *Chem Mater* **2012**, *24*, 4363-4370; f) C. Zhang, Y. Xu, M. Zhou, L. Liang, H. Dong, M. Wu, Y. Yang, Y. Lei, *Adv. Funct. Mater.* **2017**, *27*, 1604307; g) Y.-H. Zhu, X. Yang, D. Bao, X.-F. Bie, T. Sun, S. Wang, Y.-S. Jiang, X.-B. Zhang, J.-M. Yan, Q. Jiang, *Joule* **2018**, *2*, 736-746; h) Y. Chen, W. Luo, M. Carter, L. Zhou, J. Dai, K. Fu, S. Lacey, T. Li, J. Wan, X. Han, Y. Bao, L. Hu, *Nano Energy* **2015**, *18*, 205-211; i) Z. Jian, Y. Liang, I. A. Rodríguez-Pérez, Y. Yao, X. Ji, *Electrochem. Commun.* **2016**, *71*, 5-8; j) Z. Jian, Z. Xing, C. Bommier, Z. Li, X. Ji, *Adv Energy Mater* **2016**, *6*, 1501874; k) Z. Jian, S. Hwang, Z. Li, A. S. Hernandez, X. Wang, Z. Xing, D. Su, X. Ji, *Adv. Funct. Mater.* **2017**, *27*, 1700324; l) I. Sultana, M. M. Rahman, T. Ramireddy, Y. Chen, A. M. Glushenkov, *J Mater Chem A* **2017**, *5*, 23506-23512; m) W. Zhang, W. K. Pang, V. Sencadas, Z. Guo, *Joule* **2018**, *2*, 1534-1547; n) K. Share, A. P. Cohn, R. Carter, B. Rogers, C. L. Pint, *Acs Nano* **2016**, *10*, 9738-9744.

[12] Y. Qiao, S. Ye, *J. Phys. Chem. C* **2015**, *119*, 12236-12250.

[13] L. Qin, N. Xiao, J. Zheng, Y. Lei, D. Zhai, Y. Wu, *Adv Energy Mater* **2019**, *9*, 1902618.
